# Developing Medication Reviews to Improve the Aruban Healthcare System: A Mixed-Methods Pilot Study

**DOI:** 10.3390/pharmacy12040108

**Published:** 2024-07-12

**Authors:** Minke L. Copinga, Ellen A. Kok, Anke J. J. van Dam, Anoeska Wever, Adrienne Tromp, Herman J. Woerdenbag

**Affiliations:** 1 Pharmacy Master Programme, School of Science and Engineering, University of Groningen, Antonius Deusinglaan 1, 9713 AV Groningen, The Netherlands; minkelouisecopinga@gmail.com (M.L.C.); ellen.kokx@hotmail.com (E.A.K.); 2 Pharos, Expertise Center on Health Disparities, Arthur van Schendelstraat 600, 3511 MJ Utrecht, The Netherlands; a.vandam@pharos.nl; 3 Botica di Servicio, Caya Punta Brabo 17, Oranjestad, Aruba; awever@boticadiservicio.com; 4 Department of Pharmaceutical Technology and Biopharmacy, Groningen Research Institute of Pharmacy (GRIP), University of Groningen, Antonius Deusinglaan 1, 9713 AV Groningen, The Netherlands

**Keywords:** Aruba, community pharmacy, general practitioner, healthcare, medication review, patient, pharmacist

## Abstract

This study investigated whether and how medication reviews (MRs) conducted by pharmacists and general practitioners (GPs) with patient involvement can be performed on the island of Aruba (Dutch Caribbean). In this mixed-methods pilot study (both qualitative and quantitative), constructive and observational methodologies were combined. Healthcare providers’ and patients’ views on MRs and aspects of Aruban healthcare and culture relevant to MRs were examined. These insights were used to develop a protocol for conducting and implementing MRs in Aruba. Surveys were distributed and semi-structured interviews were held among Aruban community pharmacists and GPs, and a pilot program was created in which MRs were carried out with four Aruban patients and their GPs. According to the included healthcare providers, the main purpose of MRs is to optimize the patient experience and achieve concordance. Even though pharmacists and GPs consider their partnership equal, they have different views as to who should bear which responsibility in the MR process in matters regarding patient selection and follow-up. Common Aruban themes that were mentioned by the healthcare providers and deemed relevant for conducting MRs included behaviour/culture, healthcare, lifestyle, and therapy compliance. Anamnesis should be concise during the MR, and questions about medication storage, concerns, beliefs, and practical problems, as well as checks for limited health literacy, were considered important. In the pilot, at least three to, maximally, eight pharmacotherapy-related problems (PRPs) were detected per MR consultation, such as an incorrect dosage of acetylsalicylic acid, an inappropriate combination tablet for blood pressure regulation, and the absence of important laboratory values. All patients considered their consultation to be positive and of added value. In addition, it was observed that an MR can potentially generate cost savings. The information obtained from the healthcare providers and patients, together with the basic principles for MRs, as applied in the Netherlands, led to a definitive and promising MR format with practical recommendations for community pharmacists in Aruba: in comparison with the Dutch MR approach, GPs and pharmacists in Aruba could collaborate more on patient selection for MRs and their follow-up, because of their specific knowledge regarding the medications patients are taking chronically (pharmacists), and possible low levels of health literacy (GPs). Taking into account the Aruban culture, pharmacists could ask extra questions during MRs, referring to lifestyle (high prevalence of obesity), readability of medication labels (limited literacy), and herbal product use (Latin American culture). GPs and medical specialists sometimes experience miscommunication regarding the prescription of medication, which means that pharmacists must carefully take into account possible duplicate medications or interactions.

## 1. Introduction

A medication review (MR) is a method by which a pharmacist analyses the medication list and situation of a patient, discusses them with the patient’s GP and advises about possible adjustments to fit the patient’s situation and preferences. MRs aim to optimise a patient’s pharmacotherapy treatment, increasing self-management and preventing the worsening or development of conditions and complications [1]. For an MR, a community pharmacist; a general practitioner (GP); in many cases, a patient; and sometimes a geriatric or medical specialist collaborate, in order to critically evaluate the pharmacotherapy based on their clinical, pharmaceutical, and personal points of view [1,2]. An MR needs to be carried out periodically, such as on an annual basis, because a patient’s adherence to therapy may decrease, adverse events may arise, new diseases may develop, metabolism may decline, new medications may be prescribed, and new guidelines may emerge. All of these factors can alter the efficacy and safety of medicines, especially in elderly patients and in the vulnerable [1].

The greatest threats facing patients are hospital admissions and mortality due to drug use; in the ‘Integrated Primary Care Information’ (IPCI) and Hospital Admissions Related to Medication (HARM) studies, it was found that, respectively, 29% [3] and 46% [4] of the drug-related hospital admissions in the Netherlands were avoidable according to international criteria [5]. Internationally, drug-related hospital admissions have also been identified as a major problem [6]. Based on the IPCI and HARM studies, the HARM-Wrestling report contains several recommendations aiming to reduce the number of drug-related hospital admissions [5]. In the Netherlands, these recommendations are being followed, among other changes, by carrying out MRs in the community pharmacy [1,7].

In several other countries, including Australia, Canada, Chile, Germany, the United Kingdom (UK), and the United States (US), MRs are performed as well—mostly under different names, such as ‘clinical medication review’ and ‘medication assessment’ [2,8]. In each country, MRs are set up differently, with, e.g., different inclusion criteria, different divisions of labour and communication between healthcare providers, the inclusion (or not) of patient interviews, and different locations—e.g., in a community pharmacy or general practice, or at the patient’s home [1,2,8,9].

MRs in the Netherlands are structured by the method ‘Systematic Tool to Reduce Inappropriate Prescribing’ (STRIP) [7]. STRIP starts with the preparation phase, during which the pharmacist collects the patient’s data. Next, pharmacotherapeutic anamnesis takes place: the pharmacist asks the patient about their medication use and the patient’s wishes and goals when it comes to medication. Based on the patient data (including recent laboratory values) and the anamnesis, the pharmacist can start the pharmacotherapeutic analysis. At this stage, the pharmacist identifies pharmacotherapy-related problems (PRPs), such as under- and overtreatment, incorrect dosages, or potential adverse events. The pharmacist discusses the findings with the GP, and together they draw up a pharmacotherapeutic treatment plan (PTP). The final PTP is based on the prevention of PRPs and on the patient’s wishes. Subsequently, the PTP is discussed with the patient, and it is determined whether the patient has understood the information (monitoring of health literacy). After the patient has started with the interventions included in the PTP, a follow-up is carried out after a certain time to evaluate the effects of the relevant interventions [7].

MRs can have a diverse range of positive effects on patient care in different settings. Therefore, a variety of outcomes and contexts have been studied in the grey literature. In clinical pharmacy settings, studies have demonstrated an association, in the Netherlands and Australia, between MRs and a reduction in hospital visits [10,11]. In Australia, it is also recognised that MRs can offer important public health benefits [11]. However, evidence of the overall positive effects of MRs across settings appears to be scarce and sometimes inconclusive. Studies demonstrating an MR’s ability to increase therapy adherence and reduce inappropriate prescriptions and drug-related problems are the most substantial and consistent element of the literature. As to effects on mortality and morbidity, or in terms of the patient’s quality of life, findings are limited and conflicting, but there are some positive results addressing a decrease in the number of fall incidents among patients, and improvement of laboratory values such as HbA1c, blood pressure, and low-density lipoproteins (LDL). In line with this, studies on the cost-effectiveness of MRs can also be considered to be ambiguous and scarce [8].

To improve pharmaceutical care on the Caribbean island of Aruba, community pharmacies have sought to reach out to outpatients in the form of a medication review (MR). Currently, community pharmacists in Aruba perform medication monitoring, which pertains to checking prescriptions, kidney function, contraindications, interactions, and laboratory values, as well as determining whether the prescribed medication is correct for the patient. The pharmacists do not see the patient and therefore do not know the patient’s situation well.

Due to its colonial past, Aruba has a strong connection with the Netherlands. Since 1986, Aruba has been an autonomous, constituent country of the Kingdom of the Netherlands [12,13]. A reasonable number of healthcare providers on the island grew up and studied in the Netherlands, a factor which has influenced the healthcare system in Aruba, together with influences from native and Latin American healthcare providers.

Regarding demographics, Aruba is an island in the southern Caribbean Sea. Its area is 180 square kilometres, and it had a population of approximately 106,000 people in 2023. The mean life expectancy is 76.9 years. This number has been rising since measurements began in 1950 [14]. The healthcare system in Aruba consists of three medical centres, of which one is the general hospital (the Dr. Horacio Oduber Hospital). If a patient cannot be treated for a specific illness, the general hospital offers ambulance flights to an American, Colombian, or Dutch hospital. However, it may be the case that, in these countries, a patient is not insured for treatment costs [15]. There are different general practices and pharmacy practices on the island providing primary care. Although there is some variance between days, one of the pharmacy practices on the island is generally open 24 h per day. Aruba counts one health insurance company providing regulated insurance for patients: the Algemene Ziektekosten Verzekering (AZV; General Health Insurance).

Medication is imported into Aruba from suppliers in the Netherlands and the United States, as well as by commercial agents operating as brokers in Aruba itself. The commercial agents purchase medications from the factories of pharmaceutical companies. The agents are held accountable by the Aruban health authorities for this practice. The Aruban registration committee for the admission of medicines mainly registers medicines that are also registered by the European Medicines Agency (EMA). In addition, the committee adheres to the advice of the Dutch Medicines Evaluation Board (MEB). In this way, the agents are, for example, allowed to import medication from a pharmaceutical company’s factory in Colombia.

When MRs are to be performed in Aruba, the approach preferably matches with the Aruban healthcare system and culture. According to the literature, pharmacies function in universal ways, rather independent from the healthcare systems or countries they are situated in [16], which may form a gap. Furthermore, it is crucial for pharmacists to communicate with different stakeholders, such as patients, GPs, and health insurers, when MRs are to be implemented [16].

When considering how MRs can be implemented in Aruba, we take into account the healthcare system and the country (e.g., cultural themes) and show the different perspectives of patients, GPs, and pharmacists as stakeholders. For example, Aruban patients may look differently at their healthcare professionals, compared to Dutch patients. Furthermore, the Aruban climate, being much warmer than that of the Netherlands [17], could lead to other, potentially more important, recommendations for storing medication. Additionally, the Latin American culture, of which Aruba is part, is more inclined to use herbal products for health purposes. This requires more involvement of pharmacists checking possible interactions of conventional medication with herbal products.

When specifically looking at healthcare systems, the existing research has already revealed possible points for improvement on the question of how MRs could be performed. For example, US health insurance interests and a number of GPs in New Zealand would recommend having a pharmacist perform the MR in the context of a general practice. German GPs would like to select the patients themselves, instead of having this performed by the pharmacists. According to them, this would ensure that patients would more quickly agree with advice given them on medication adjustments resulting from an MR. Pharmacists from different countries have indicated that they wanted more access to patients’ medical data in order to perform their MRs more efficiently. Furthermore, GPs find it very important to receive short reports, rather than long documents that they have to work through. In general, pharmacists and GPs should strive for a positive working relationship with mutual respect [16]. Such nuances are also found in the Aruban situation and are taken into account when discussing the implementation of MRs in Aruba.

This study aimed to investigate whether the implementation of MRs according to the Dutch methodology could ‘fit in’ in the context of the Aruban healthcare system, and to identify which adaptations are needed to the MR strategy in order to create a better match with Aruba. A protocol was developed for conducting MRs which was based on the Dutch MR setting. Subsequently, through interviews, GPs, pharmacists, and the pharmacy owner of Botica di Servicio Aruba gave their views on MRs in the context of Aruba. In addition, a pilot program of MRs with Aruban patients was carried out to evaluate the ways in which patients, pharmacists, and GPs could encounter issues within the process and how these could be solved. The final goal was to design an effective and efficient protocol for carrying out MRs in Aruba, fitting into the Aruban healthcare system and culture and contributing to improved pharmaceutical patient care.

## 2. Materials and Methods

### 2.1. Design and Participants

**Figure 1:**
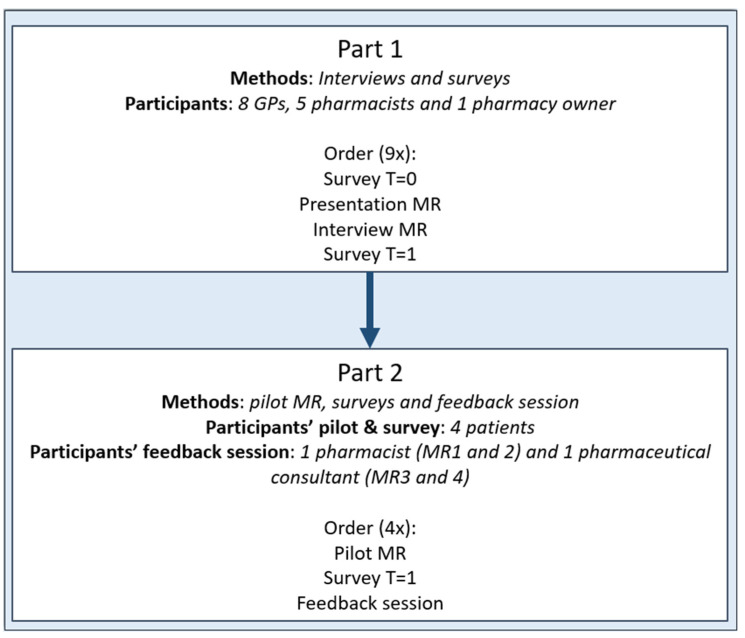
Design of the mixed-methods pilot study on implementing medication reviews in Aruban pharmaceutical healthcare.

In this mixed-methods study, a term which signifies a study with qualitative and quantitative aspects, constructive and observational methodologies were combined. The reason for choosing this approach was to integrate insightful comments from healthcare providers and patients about MRs with quantitative information about PRPs. The study was conducted on the island of Aruba in May–June 2023. The study was initiated by an assembly of community pharmacies called ‘Botica di Servicio Aruba’, in collaboration with researchers from the Netherlands. Botica di Servicio is one of the chains of community pharmacy practices on the island. The study consisted of two components. In part 1, interviews and surveys were conducted with healthcare providers. In part 2, a pilot study was carried out with patients. An overview of the study’s setup is provided in Figure 1.

The interviews and surveys on the views of MRs in Aruba in the first part of the study were conducted with eight GPs, five pharmacists, and one pharmacy owner. The pharmacy owner does not have a pharmaceutical background. He focuses on the business operations of all pharmacies of Botica di Servicio in Aruba. Therefore, in the context of conversations with the pharmacy owner, it was it is important to discuss and make clear agreements about future plans. The pharmacists included were the clients of the study: they wanted to see if MRs could work out well in Aruba. The GPs had to speak English or Dutch (so that M.L.C. and E.A.K. would be able to talk to them) and had to have collaborated with Botica di Servicio in Aruba. In practice, all GPs in Aruba collaborate with all pharmacies on the island.

The interviews were semi-structured. Interviews with the GPs were scheduled one-on-one on different days. Interviews with the pharmacists and the pharmacy owner were scheduled as one encounter, as a group. The pilot of MRs in the second part of the study was carried out with four Aruban patients. MR1 and MR2 were carried out on different days. MR3 and MR4 were carried out consecutively on the same day. The feedback sessions connected to the pilot were held either with one pharmacist (for MR1 and MR2), or with one pharmaceutical consultant (MR3 and MR4) of Botica di Servicio Aruba, depending on who was attending the pilot MR consultation. The contents of the interviews and surveys, together with the points of interest drawn up during and after the pilot, were used to make recommendations on how to set up and implement MRs in Aruba.

The included GPs work in primary care general practices across Aruba; the study thereby covers all areas in Aruba. The five pharmacists and the pharmacy owner all work at Botica di Servicio Aruba. The four patients were care recipients associated with two GPs who work in two different general practices in Aruba, and who were also included in the first part of the study. These GPs were selected for the pilot, as they were open to participating with the pharmacists in the context of the pilot. See Table 1 for a description of the study population with respect to the healthcare professionals and patients. The pharmacy owner is a Dutch male who has a business degree and who has been working for nine years as a managing director for the indicated pharmacy organisation. The obtained sample sizes representing the different stakeholder groups and their composition are the result of convenience sampling.

#### 2.1.1. General Approach Part 1—Interview Healthcare Providers

During part 1 of the study, an introductory presentation by E.A.K. about MRs was given to the included healthcare providers. An interview (see Section 2.2.2.) was held after the presentation, in order to obtain feedback on how best to implement MRs in Aruba, taking into account the situation of Aruban healthcare. Specifically, the presentation provided a general introduction to the concept and (STRIP) execution of MRs according to the Dutch standards formulated by the Royal Dutch Pharmacists Association (KNMP) [1]. For the interviews, the first version of a protocol for conducting MRs in Aruba was designed by M.L.C. (see Supplementary Materials, Overview S1). This version was based on the STRIP method and previously acquired knowledge about Aruban healthcare and the inhabitants of Aruba. The extent of knowledge about MRs and the motivation of healthcare providers to start conducting MRs in Aruba were assessed using a survey. The same survey was used twice, i.e., before and after the interview (see Supplementary Materials, Overview S2).

#### 2.1.2. General Approach Part 2—Pilot Medication Reviews

During part 2, two GPs were approached in order to see if they knew patients who they thought might be appropriate candidates for an MR; this would mean that these patients might have (several) pharmacotherapy-related problems (PRPs) because of polypharmacy, and on the other side, were open to speaking about their medical situation. After agreement was reached as to the patient selection, these patients were invited by a pharmacist or a pharmaceutical consultant of Botica di Servicio Aruba to take part in the pilot. Subsequently, the medical histories of these patients were collected, as documented in the GP system, important lab results, and medication overviews. Three of the cases were prepared by M.L.C. and one by E.A.K. After this, the MR consultations were conducted by M.L.C. and E.A.K. together, each taking the lead with ‘their own’ patient(s). One MR was conducted in Dutch, one in Dutch/Papiamento (translated by a pharmacist into Dutch when needed), one in English, and one in Spanish/Papiamento (translated by a pharmaceutical consultant into Dutch). After each MR, data on patient experience were collected through a survey (see Supplementary Materials, Overview S3). The first MR was carried out with an initial version of the protocol, as mentioned in Section 2.1.1. After each MR, this protocol was adjusted based on feedback sessions with researchers M.L.C. and E.A.K., together with a pharmacist or the pharmaceutical consultant from Botica di Servicio Aruba. For all of the patients, a pharmacotherapeutic analysis was carried out to define PRPs. Possible interventions related to the PRPs were discussed with the GPs of the patients, after which definitive PTPs were made. All of the patients were then severally called to inform them about their PTPs, and upon agreement, a date was planned for follow-up. Follow-up took place after approximately two weeks via phone calls. In addition, a cost–benefit analysis was performed on the pilot MR consultation that yielded the most medication policy interventions.

### 2.2. Data Collection

#### 2.2.1. Part 1, Surveys of GPs and Pharmacists

Regarding the survey, two moments of data collection from GPs and pharmacists were included in the study: T = 0 was measured before the presentation and interview about the MRs; T = 1 was measured after the presentation and interview on the MRs. The content of the survey consisted of multiple choice, open-ended, and Likert-scale questions and was the same for both data collection moments. The questions asked in the surveys were based on the studies of Elyan et al., Smith et al., and Jahangirian et al. (see Supplementary Materials, Overview S2) [18,19,20].

#### 2.2.2. Part 1, Interviews of GPs, Pharmacists, and Pharmacy Owner

After the presentation about MRs was given, the GPs, pharmacists, and the pharmacy owner were interviewed while going through the first version of the protocol together (see Section 2.1.1.). The healthcare providers spoke about their vision for the MRs in the context of Aruba and provided feedback on the protocol.

#### 2.2.3. Part 2, Pilot MR

Following each MR consultation, a feedback session was carried out to discuss and process the patient’s quotes, proposed interventions, and points of interest for the subsequent MRs.

#### 2.2.4. Part 2, Survey Pilot MR

For the patients participating in the pilot, there was one data collection moment: T = 1, which took place after the MR. The content of the survey was based on the Generic Short Patient Experiences Questionnaire (GS-PEQ) by Sjetne et al. and only consisted of Likert-scale questions; see Supplementary Materials, Overview S3 [21]. Survey versions were available in two languages: Dutch and English. Both survey versions contained a smiley-face rating scale to support the answer possibilities. Patients received the survey version associated with their preferred language.

#### 2.2.5. Ethical Considerations

Before the patients were included in the pilot, all patients were asked to sign informed consent forms after having been provided a comprehensive explanation in lay language (level A2/B1) of the content and purpose of the study. Through informed consent, patients agreed that their input could be used for the purposes of elaboration of the study results and possible publication. Patients could withdraw from the study at any time without a reason. Informed consent was also obtained from all included healthcare providers (GPs and pharmacists) and the pharmacy owner.

### 2.3. Data Analysis

#### 2.3.1. Analysis of Surveys

The answers obtained to the Likert-scale questions were elaborated by calculating the median. The responses to the Likert-scale questions were assigned values in ascending order, starting at 1 and in intervals of 1. For the 5-point scale, used for most questions, this means: ‘Strongly disagree’ or ‘Very unlikely’ = 1, ‘Disagree’ or ‘Unlikely’ = 2, ‘Neutral’ = 3, ‘Agree’ or ‘Probably’ = 4, and ‘Strongly agree’ or ‘Very likely’ = 5. A similar ranking was applied to the 4- and 7-point Likert scales used to assess one question each in both pharmacist and GP surveys. For the responsibility percentages, the average of the answers for each stakeholder group was calculated, while the MR main-purposes percentages represent the share relative to the total number of participants in each stakeholder group.

#### 2.3.2. Analysis of Interviews

After each interview, the topics discussed were documented and codes that were connected to one of the themes shown in Table 2 were associated with the data. For the protocol MR, coding was not the most effective way of documenting the results, as the input was based on all the different recommendations obtained from the healthcare professionals. Therefore, the points of interest were summarized and divided into ‘pharmaceutical history’ and ‘carrying out an MR’.

#### 2.3.3. Analysis of Pilot MR

The topics discussed during the feedback sessions after each MR were elaborated based on quotes, quantitative data, and summaries. These approaches were more useful, compared to coding. Because there were very few patient reactions, only quotes were used. More insight into the interventions of MRs was obtained by counting them, forming a graphical overview. As for the elaboration of MRs it was decided to summarise the findings, as too many different codes would have been needed. Table 3 provides details about the data processing. Further, the monetary value in the cost–benefit analysis was calculated based on the average life expectancy of inhabitants in Aruba, which is estimated to be 77 years of age [22], and the at-the-time-valid medication prices corresponding to the medication policy interventions. To derive the total costs of the MRs’ proposed changes in medication policy, total medication costs of the medication policy before and after the MR were calculated based on import prices (valid for June 2023) and the difference was subsequently determined. This monetary value was then adjusted based on the remaining period of the average life expectancy. Initially, this value was expressed in Aruban Florin (AWG), and it was converted to Euros (EUR) and US Dollars (USD) based on the currency exchange rates valid during the period of the proposed change.

## 3. Results

### 3.1. Healthcare Providers’ Views on MRs

#### 3.1.1. Main Purposes of Conducting MRs

**Figure 2:**
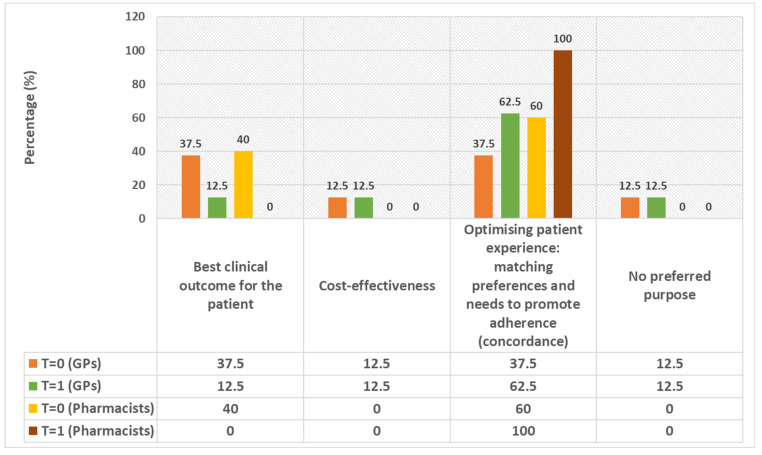
The main purposes of an MR, according to GPs and pharmacists, measured before education about MRs (T = 0) and after education about MRs (T = 1). The shares of the different answers are shown in percentages (%).

Figure 2 shows the main purposes of the MRs according to GPs and pharmacists. In particular, two themes were identified before the presentation about MRs: ‘best clinical outcome for the patient’ and ‘optimising patient experience’. After the education moment, the opinion of most GPs (62.5%) and pharmacists (100%) shifted to the theme of ‘optimising patient experience’ as being the main purpose of an MR.

#### 3.1.2. Attitude towards and Knowledge about MRs

**Figure 3:**
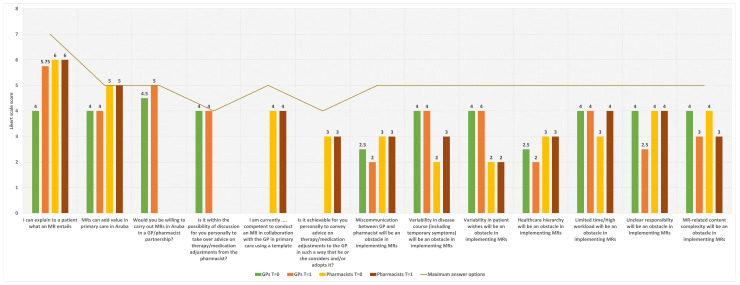
Attitude towards and knowledge about MRs of Aruban GPs and pharmacists, measured before education about MRs (T = 0) and after education about MRs (T = 1) in terms of the scores on the Likert-scale questions. The number of maximum answer options per question is represented by the brown line.

Figure 3 shows the Likert-scale scoring on the questions addressing the attitude towards and knowledge about MRs held by GPs and pharmacists. GPs strongly believe that MRs add value to primary care in Aruba. Furthermore, the GPs indicate that they are always willing to take advice on possible adjustment of medication from the pharmacist. As with GPs, pharmacists strongly believe that MRs add value to primary care in Aruba. Furthermore, they believe that they are sufficiently good at explaining to a patient what an MR entails. Pharmacists also feel that they are sufficiently competent to perform an MR in the context of primary care in Aruba, noting that they would often be able to properly convey advice on possible adjustments of medication to the GP.

The training moment affected GPs’ perspectives on several aspects of MRs. First of all, GPs indicated that the training moment improved their ability to explain to a patient what an MR entails. Furthermore, the willingness to participate in an MR pilot with Aruban pharmacists increased slightly. Moreover, the training event caused GPs to see fewer obstacles in implementing MRs. In particular, unclear responsibility was seen as less of a problem than initially thought. Among the pharmacists, the training moment mainly influenced their assessments of impeding factors in the implementation of MRs. For instance, after the training moment, pharmacists believed that variability in disease course and limited time/high workload would pose greater obstacles than initially thought. In contrast, as a result of the training moment, they expected that the content complexity of MRs would be less than previously thought. After the training moment, all GPs and pharmacists indicated that Aruban healthcare providers would benefit from training on the subject.

#### 3.1.3. Opinions about Responsibilities while Conducting MRs

**Figure 4:**
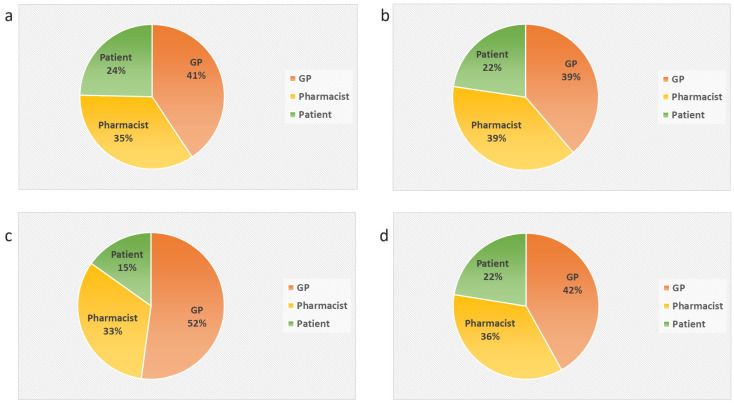
Overview of the opinions about the overall responsibility while conducting an MR. Average percentages are shown that reflect the degree of responsibility of the GP, pharmacist, and patient in an MR, as described by the participating pharmacists and GPs. (**a**) Opinions of the pharmacists before the education about MRs (T = 0). (**b**) Opinions of the pharmacists after the education about MRs (T = 1). (**c**) Opinions of the GPs before the education about MRs (T = 0). (**d**) Opinions of the GPs after the education about MRs (T = 1).

Figure 4 shows the distribution of the responsibility percentages per stakeholder group, as formulated by GPs and pharmacists. Both pharmacists and GPs initially gave the greatest responsibility for conducting MRs to GPs (41–52%), followed by pharmacists (33–35%), and finally patients (15–24%). After education about MRs, the partnerships between GPs and pharmacists in terms of responsibility within an MR became more equal (~40%).

#### 3.1.4. Responsibility for Patient Selection before Conducting MRs

During the interviews, GPs, pharmacists, and the pharmacy owner of Botica di Servicio Aruba gave their views on task delegation during the conduct of MRs. In particular, GPs shared their opinion on who should carry out patient selection, while the pharmacists did not provide an opinion on this aspect. The GPs gave different views: one GP indicated that the general practice-based nurse specialist can select patients from the general practice. Three GPs would have liked to have the pharmacist carry out the patient selection, of which two GPs stated that setting a deadline for patient selection is useful. One GP mentioned, for example, that it is useful to discuss the patient selection once every two months. The GPs’ justification for having the pharmacist take the lead in the patient selection was that the pharmacists often have a better overview of the medication in use. This is partly because patients can change between GPs twice a year, resulting in GPs having a poorer medication overview. Another GP indicated that both the GP and the pharmacist can select patients and then discuss the patient selection once every two months.

#### 3.1.5. Responsibility for Follow-Up after Conducting MRs

The pharmacists commented that the GP is best able to handle the follow-up, because, according to the pharmacists, the patient probably is more accepting of information from the GP. The pharmacists also noted that it is better to have the patient come physically to the general practice instead of the GP calling the patient. A physical appointment allows the GP to better assess how the patient has dealt with the interventions and what the best plans are for the patient in the future. Indeed, a GP mentioned, just like the pharmacists, that the GP can take the lead in the follow-up. Meanwhile, another GP mentioned that both the GP and the pharmacist can perform the follow-up, with the help of the general practice-based nurse specialist. Additionally, a different GP noted that pharmacists can perform the follow-up, after which they can set up the conclusions and email them to the GP to update the patient file.

#### 3.1.6. Main Challenges in Implementing MRs

**Figure 5:**
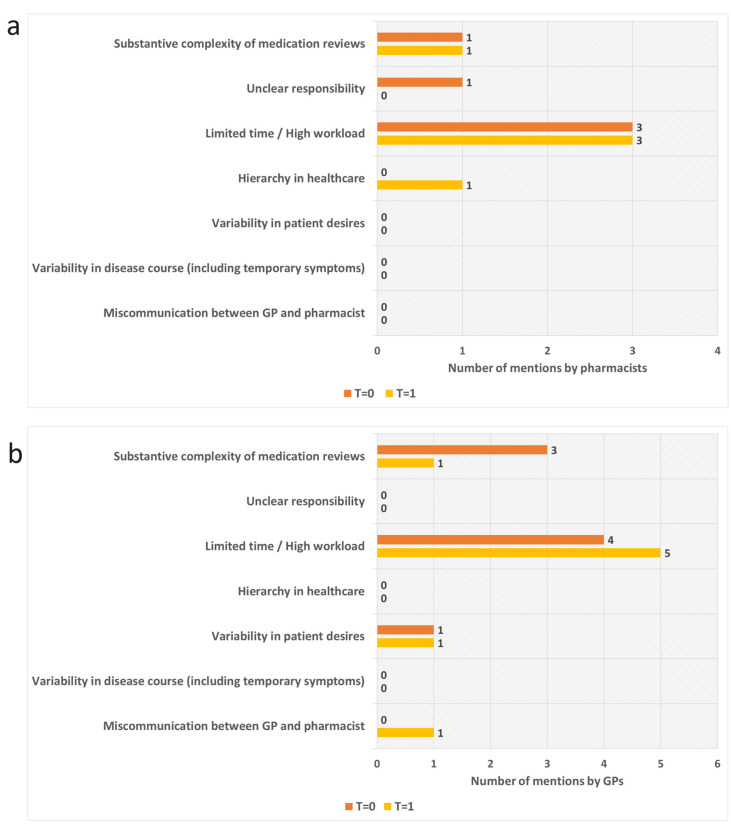
Description of main impediments in conducting MRs, according to the participating GPs and pharmacists. Each GP and pharmacist was only allowed to indicate the one impediment which they considered most relevant. (**a**) Opinions of the pharmacists before education about MRs (T = 0) and immediately after education (T = 1). (**b**) Opinions of the GPs before education about MRs (T = 0) and immediately after education (T = 1).

Figure 5 shows the expected main challenges in implementing MRs in Aruba according to pharmacists and GPs, indicated via an open-ended question in the survey. Limited time/high workload was seen as the main obstacle. However, three pharmacists and three GPs indicated in the explanatory note that miscommunication is in a close second place, as it also has substantial potential to impede cooperation. All of the GPs and one pharmacist indicated that miscommunication is mainly caused by the unclear assignment of responsibilities during the process, as MRs encompass a multidisciplinary approach. However, according to the other two pharmacists, miscommunication is mainly caused by hierarchy in healthcare (in which the GP holds a more important role in the patient relationship, in comparison with the pharmacist).

### 3.2. Themes in Aruba

**Figure 6:**
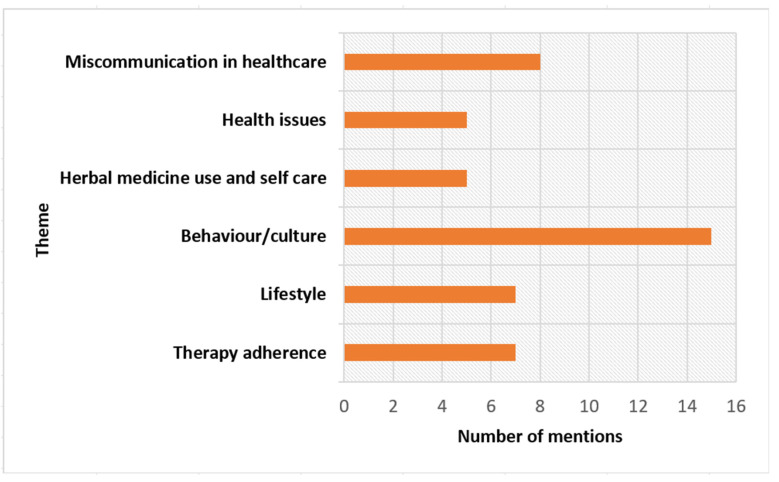
Overview of themes that may play a role during MRs in Aruba, according to the participating GPs, pharmacists, and pharmacy owner.

According to their experiences, GPs, pharmacists, and the pharmacy owner talked about various themes that could influence an MR or could play a role during an MR in Aruba. The number of times these themes were mentioned by healthcare providers is shown in Figure 6. The most frequently mentioned themes were related to behaviour/culture, miscommunication in healthcare, lifestyle, and therapy adherence. The other topics mentioned were health issues, such as excessive NSAID use, addiction to sleep medication, and under-treatment, in addition to the extensive use of herbal products. In the area of behaviour/culture, healthcare providers argued that there are particular differences between the Latin American and Dutch cultures in the field of healthcare, which might imply that MRs with patients from different cultures require different approaches. Furthermore, the healthcare providers mentioned therapy adherence as a significant theme, which points specifically to non-adherence. According to healthcare providers, possible causes of non-adherence relate to a low level of education, shame, multilingualism, intellectual disability, and limited health literacy. Regarding lifestyle, we noted that diabetes, being overweight, and obesity are highly prevalent in Aruba.

### 3.3. Content of Aruban MRs

#### 3.3.1. Patient Selection

Pharmacists preferred to select patients starting from the age of 40 because diabetes mellitus type 2 and myocardial infarctions can already begin to happen around this age. Three GPs shared this opinion, of which one mentioned that younger people can benefit more from lifestyle interventions, and another GP mentioned that the quality of life of younger patients can be improved more than the quality of life of older patients. Furthermore, healthcare providers consider it particularly important to conduct a review for diabetes and dementia patients, as these diseases are considerably prevalent in Aruba. They also think it is important to include elderly patients in MRs, for which some GPs mentioned a preference for including patients older than 50 or 70 years of age, while other GPs did not give a specific age indication. One GP pointed out that older patients can be eligible for deprescribing. Two GPs noted that it is more important to look at the number of medicines a person is taking in comparison to their age. A GP also specifically stated that the number of comorbidities is important to consider. Subsequently, one GP suggested selecting patients based on an increase in the required number of medicines with the decreasing ages of potential candidates—for example, 80 years of age and five medications and 70 years of age with six medications.

#### 3.3.2. Anamnesis

The healthcare providers gave their responses concerning the implementation of an MR as a whole. The responses in Table 4 address the following areas: ‘inviting patients’, ‘anamnesis’, ‘drawing up a treatment plan’, and ‘follow-up’.

The healthcare providers also specifically gave their responses concerning the anamnesis. The responses in Table 5 address the following areas: ‘medication’, ‘complaints’, ‘practice’, and ‘lifestyle’.

### 3.4. Pilot MRs

#### 3.4.1. PRPs Obtained 

**Figure 7:**
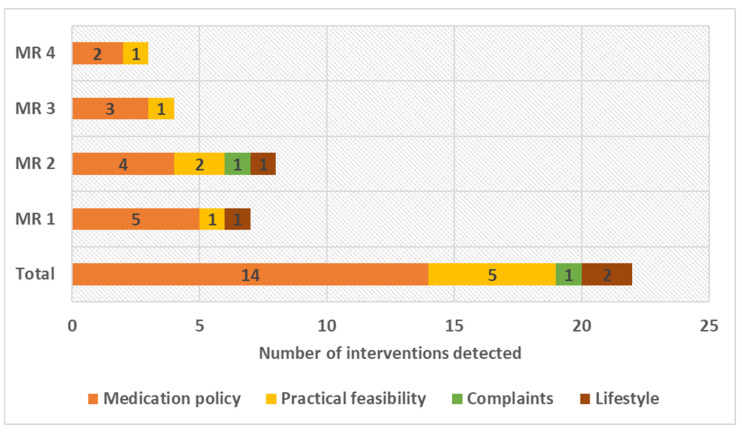
The number of interventions detected during the pharmacotherapeutic anamnesis of the pilot MRs.

Figure 7 shows the number of PRPs that were detected per pilot MR and in which areas the interventions were related. In the pilot, a minimum of three and a maximum of eight PRPs were detected per consultation. The largest proportion related to medication policy, followed by practical feasibility, lifestyle, and complaints.

In MR1, which concerned a 72-year-old patient, a cost–benefit analysis was also conducted. In this MR, five PRPs related to the medication policy were detected: occurrence of dual platelet aggregation inhibition therapy, incorrect dosage of acetylsalicylic acid, inappropriate combination tablet for blood pressure regulation, ineffective statin treatment due to high LDL cholesterol, and the absence of important laboratory values. The cost–benefit analysis showed that the proposed interventions in the PTP could have the potential to save 3707.40 AWG (1889.42 EUR/2058.29 USD). At the end of the pilot (week 26, June 2023), an amount of 2954.40 AWG (1505.67 EUR/1650.24 USD) was already saved, as most proposed interventions, i.e., four out of five, were implemented directly. See Table 6 for an overview of the calculations performed.

#### 3.4.2. Patients’ Opinions

**Figure 8:**
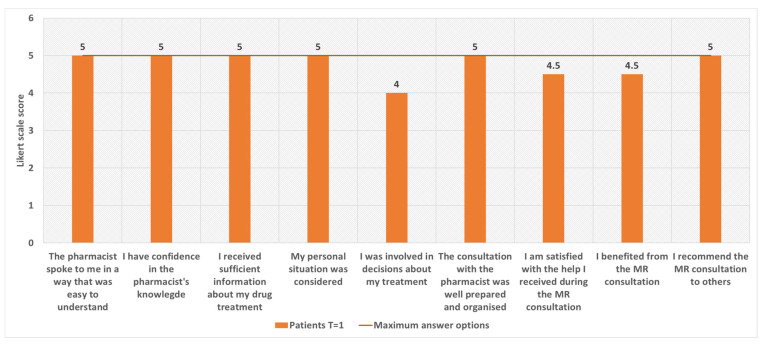
The experiences of the patients participating in the pilot MR, measured by means of a survey. Experiences are displayed by means of a Likert-scale score. The number of maximum answer options per question is represented by the brown line.

Figure 8 summarises the experiences of patients who participated in the pilot, as measured by a survey (see Supplementary Materials, Overview S3). All survey questions/statements were scored as satisfactory, with six out of nine being assigned the highest score. For example, patients felt that the pharmacist spoke to them clearly, was adequately knowledgeable, and was well-prepared for the consultation. The patient’s personal situation was also taken into account and the patient received sufficient information. As a result, all patients would recommend an MR to other residents of Aruba. The statement that received the lowest score (score 4/5), even though it still appears to be positive because it means ‘agree’, indicates that the greatest improvement potential is in the area of patient involvement in decision-making about their drug treatment.

#### 3.4.3. Specific Points of Interest

After each MR, the protocol for carrying out an MR was adapted based on the process of the pilot. One goal was to make the protocol succinct. The different subsequent versions and the final draft of the protocol, which was based on the first version, are shown in Supplementary Materials, Overview S4. The various comments noted after each MR during the pilot are shown in Table 7.

## 4. Discussion

This study reveals insights on implementing MRs in Aruba and adjusting them towards the Aruban culture and healthcare system. Some key highlights are the division of responsibilities and patient considerations like limited health literacy, the use of herbal products, and lifestyle diseases. Furthermore, the results of the pilot provided information about patients’ needs, important logistical agreements, and time management for MRs. All of these results gave rise to a new protocol for carrying out MRs in Aruba, and these findings and consequent recommendations will offer possibilities for future approaches in conducting MRs, with specific relevance to the Caribbean island of Aruba. This offers a new way of thinking about MR implementation, combining practical measures used to implement MRs with key aspects of a country’s healthcare system and cultural base. This approach may inspire other pharmacists to focus on a more diverse approach towards patients, taking into account their levels of health literacy when conducting an MR. If MRs are not yet being conducted in a country, our study may help such a country implement MRs appropriately, by fitting them with the local healthcare system and cultural situation. In this way, pharmaceutical care can be improved. For example, focused efforts are being made to improve the quality of pharmaceutical care in Aruba, which led to Botica di Servicio being certified in 2022 according to Dutch guidelines for pharmaceutical care.

Several Caribbean islands could specifically benefit from this study, due to their connection with the Kingdom of the Netherlands, by following the Dutch guidelines in the pharmaceutical professional field, in a manner similar to Aruba. Bonaire, Sint Eustatius, and Saba are municipalities of the Netherlands called the Caribbean Netherlands. Likewise, Aruba, Curaçao, and Sint Maarten are separate countries in the Kingdom of the Netherlands [12]. All islands are located in the Caribbean Sea, between North, Central, and South America [17], which underlines their ties with Latin American cultures. The situation regarding pharmaceutical care is therefore highly comparable to that in Aruba. Sint Maarten, in particular, may differ culturally from the situation in Aruba because the northern part of the island is part of the French Republic. Still, the Dutch part could benefit from the findings of this study [12].

### 4.1. Factors for Medication Adherence

To the best of our knowledge, there are no countries that take cultural differences within a country into account when implementing MRs. According to the literature, cultural differences can lower medication adherence [23]. As medication adherence is an important point of interest for MRs [1], cultural differences are a valuable factor to take into account when implementing MRs.

The literature shows that medication adherence can be influenced by cultural beliefs. For example, it has been shown that Chinese asthma patients who have low levels of medication adherence more often believe in the use of traditional herbal products for asthma, instead of conventional medication via inhalers. This may be because Chinese patients believe more in the benefits of these herbal products, and see fewer benefits from Western medication [23].

In the course of our study, differences between Latin American and Western culture became clear. On the one side, the healthcare providers were trained in the Netherlands and exposed to the Western healthcare culture, with its conventional medicines, while on the other hand, Aruban patients are often closely involved in Latin American culture, where herbal products often play a major role.

Next to the cultural differences, it was also shown that spirituality and low health literacy were significantly associated with lower medication adherence. Furthermore, knowledge and personal perceptions about an illness, as well as self-efficacy, are associated with good medication adherence [23]. From our study it appears that such knowledge is sometimes lacking in the Aruban situation. One example of limited health literacy in Aruba is connected to the package leaflets of medicines. These are often written in Dutch or English, but not in Papiamento or Spanish. The last two languages are precisely the languages that Aruban patients speak best. During MRs, a digital tool may be used (already used by Botica di Servicio), which pronounces understandable language and shows animated videos about the use of specific types of medication.

### 4.2. Healthcare Providers’ Views on MRs

Both pharmacists and GPs agreed that the main goal of an MR is to enhance the patient experience and treatment outcomes by creating a tailor-made therapy, attempting to encourage patients to adhere to their treatment. This perspective was particularly shared following education on MRs.

Education about MRs provided to GPs led them to see fewer obstacles to the implementation of MRs. The opposite was true for pharmacists, possibly due to the STRIP method, which places the pharmacist at the centre of conducting an MR rather than the GP [1,24]. Therefore, it is important that pharmacists and GPs work together to ensure that the problems pharmacists experience are properly addressed and solved.

Following training, both GPs and pharmacists would give each other about the same responsibility-based percentages for conducting an MR, implying that they consider each other to be equals in the treatment partnership. The equal partnership is also laid down in the Medical Treatment Agreement Act [25]. Equal partnership is important, as aligning resources towards an equal and common goal is a crucial aspect in facilitating stakeholder engagement during inter-organizational innovations, such as an MR [1]. In addition, equal cooperation between different healthcare providers is a prerequisite for fostering complex decision-making on the best treatment for the patient [26].

It is recommended that community pharmacists and GPs make clear agreements, focussing on responsibilities during the MR process, as the healthcare providers mentioned miscommunication and hierarchy roles in the Aruban healthcare system as potential barriers. These points also cohere with results from other studies that mention the importance of clear and efficient divisions between roles for GPs and pharmacists during MRs [16]. This is especially crucial, given the limited time and high workload challenges in healthcare [26]. To further reduce the workload, it might be worthwhile to consider having some tasks be performed by the nurse practitioner/doctor’s assistant and the pharmaceutical consultant/pharmacy technician.

In line with this, it is important to have GPs and community pharmacists properly deliberate about patient selection. While some prefer that pharmacists carry out patient selection due to their clear overview of a patient’s medication policy via the pharmacy information system, others advocate for GPs, mentioning the latter’s expertise on the patient’s disease(s) and personal situation, such as limited health literacy, lifestyle problems, or use of herbal products. Considering these points of view, collaboration is thought to give rise to the best patient selection. GPs could determine whether a patient is eligible for an MR, and pharmacists could check the pharmacy information system to identify the number of medicines associated with a patient. Ultimately, GPs and pharmacists can discuss together which patients will be invited for an MR. The same advice applies to the follow-up. To facilitate this collaboration structure, we suggest that the healthcare providers adhere to a mutually agreed schedule for patient selection. In the beginning, for example, a collaborative structure can be adopted in which six patients are selected together every two months.

### 4.3. Themes in Aruba

Healthcare providers mention behaviour/culture, healthcare practices, lifestyle factors, and therapy (non-)compliance as major themes playing roles in conducting MRs in Aruba. Striking remarks were made about the contrast between Latin American and Dutch culture, possibly leading to ‘clashes’ between the GP and patient: many Aruban patients travel to Colombia for hospital treatments, receiving ample medical check-ups, while Dutch healthcare practices prefer to adhere to a wait-and-see policy [27].

According to the healthcare providers, an important aspect surrounding Aruban therapy (non-)compliance is limited health literacy, resulting from factors such as low educational levels, multilingualism, and intellectual disabilities. This is confirmed by the high prevalence rates of limited health literacy in Aruba [28]. This underscores the importance of healthcare providers recognizing and addressing this issue in the context of MRs, and having sufficient skills to educate patients, ultimately promoting therapy adherence among patients [29,30].

Moreover, healthcare providers stressed the importance of easily understandable MRs, due to the high rate of limited health literacy. The MR protocol already contained smiley face rating scales and simplified language. An overview of the smiley face rating scales used for the MR protocol is shown in Supplementary Materials, Overviews S1 and S4.

Furthermore, according to healthcare providers, many patients in Aruba suffer from diabetes, obesity, and being overweight, pointing out that lifestyle plays a major role in conducting MRs in Aruba. Indeed, it is known from the literature that more than 75% of the population aged 20 years and older is presently overweight [31]. Lifestyle guidance in MRs could prevent lifestyle-based diseases such as cardiovascular disease, or the worsening.

Another important aspect for MRs is the seemingly highly amount of herbal products used in Aruba, according to the healthcare providers. These products are traditionally used for medicinal purposes in Latin American culture and the practices are passed on from generation to generation [32]. Since many Latin American people live in Aruba, and patients have the free choice to travel to Colombia for healthcare, this could potentially also contribute to more extensive use of herbal products. Herbal products used in Aruba are probably often not commercial products which are registered, but rather loose herbs that are bought on the market or collected from nature by people. Thus, the quality of herbal products is not guaranteed, and safety factors are not proved or documented. Therefore, the use of these products by patients should be registered by pharmacies and/or GPs in order to control their use and address possible hazardous effects such as adverse events or diminishing effectiveness of medications due to their interaction potential. Examples of herbal products that may be used by the Aruban population include oregano, aloe, chamomile, yerba bueno, moringa, and basora pretu [33].

A last point connects to miscommunication surrounding prescription medications, following healthcare providers’ stories: medical specialists and GPs do not always know what the other prescribes, which increases the risk that, for example, duplicate medications or interactions occur. Possibly, MRs can help to determine certain PRPs that have originated due to miscommunication, and which have not been identified during the process of medication monitoring.

### 4.4. Content of Aruban MRs

In the context of Aruban MRs, healthcare providers prioritise patient selection, based on the amount of medicine a person is taking, above age. As, according to the healthcare providers, limited health literacy has been mentioned as a significant problem in Aruba, this aspect should also be considered in patient selection. Therefore, this study recommends selecting patients using at least five different types of chronic medication, together with the presence or absence of limited health literacy. GPs could indicate the presence of limited health literacy, based on conversations and consultations with patients. In cases with less than five different types of medications, the occurrence of limited health literacy should be an adequate reason to include a patient in an MR. No specific age should be associated with patient selection, partly due to the views of the healthcare providers and partly due to evidence from other countries that do not presently consider age in patient selection [2,34].

A study by Rose et al. indicated that it is efficient to apply patient selection with both objective and subjective criteria [35]. For this reason, it is recommended that GPs and pharmacists jointly select patients with at least five chronically used medicines, as is the case in the Netherlands [1,7], regardless of the patient’s age. Subjective criteria may include limited health literacy and (un)conscious non-adherence (see Section 3.2.), because in general, limited health literacy and non-adherence lead to incorrect use of medicines [36] and a higher risk of PRPs.

Before the MR pilot started, the healthcare providers provided feedback during the interviews on the intended process of MRs in Aruba, based on the first version of the protocol to be used to conduct MRs (see Supplementary Materials, Overview S1). Healthcare providers recommended inviting patients by telephone or WhatsApp, as the latter is widely used on the island. Furthermore, instead of inviting patients annually for MRs, healthcare providers advised that an MR be reperformed for a particular patient every three years, as this leads to a smaller workload.

Another point concerned communicating to the patient that all outcomes will be discussed with the GP, and that pharmacists employ confidentiality, thereby promoting patient honesty. At the end of the anamnesis, it is crucial to explain what changes may occur in the medication list and to discuss how the patient feels about these proposals.

For the evaluation with the GP about the treatment plan, healthcare providers consider the BAAN form to be an efficient tool. They agreed to let the pharmacist send this form to the GP one week before the evaluation moment.

Suggestions for follow-up included collecting patients’ old medications, analysing non-verbal communication, and empowering patients to schedule their appointments. Additionally, it was recommended that the responsibility of the follow-up be delegated to the GPs.

In pharmacotherapeutic anamnesis, healthcare providers highlight the importance of assessing patients’ understanding of their medications and asking about herbal and over-the-counter remedies. Smile scales are thought to be important for assessing complaints. To look at practical barriers, healthcare providers asked patients to read labels to assess the health literacy and understanding of the patients. As for mentioning possible adverse events, healthcare providers do not think that it would be useful to list all kinds of adverse events, because patients can then determine that all of them are bothering them (nocebo).

In terms of practice, GPs and pharmacists think it is useful to ask how medication is stored. Aruba has a tropical climate, which means that medication that needs to be cooled must be kept in a refrigerator in order to maintain its shelf life. Furthermore, healthcare providers value providing instructions for, for example, inhaled medications, creams, and eye drops, and asking patients about concerns, beliefs, and any practical problems that patients may experience. In cases of practical problems, healthcare providers recommended referring to YouTube videos or Kijksluiter videos, where explanations are given about taking or using medication.

In cases of lifestyle problems, the pharmacist can refer to the GP when necessary. In addition, when questioning the amount of alcohol the patient is drinking, healthcare providers also advise that the patient be specifically asked about wine and beer. This is because not everyone in Aruba sees wine and beer as alcoholic beverages. In addition, healthcare providers debated whether it is useful to ask what someone eats every day, since unhealthy eating patterns are often influenced by poor economic status which cannot be improved immediately. Therefore, it is important to evaluate the need for this ‘lifestyle’ component in a subsequent MR pilot.

### 4.5. Pilot MRs

After each MR, the protocol was adjusted based on feedback sessions with researchers M.L.C. and E.A.K., together with a pharmacist or a pharmaceutical consultant from Botica di Servicio Aruba. Through this approach, the researchers were able to optimise the MR process gradually.

Patients appeared to have had a positive experience with their MR and therefore recommended the MR consultation to other Arubans. One reason for this enthusiasm had to do with the community pharmacist profiling itself as a healthcare provider; many patients reported having no idea what the community pharmacy does. Meanwhile, the pilot showed that patients often need the expertise of the pharmacist in terms of additional information or guidance on their medication use. This presents an opportunity for Aruban community pharmacies to raise their image as healthcare providers. This strategy could start, for example, by seeking out the media (more).

The number of interventions implemented shows that an MR adds value in Aruba, as there is a correlation between the presence of a PRP and the increased quality of life of the patient [1,37]. In our pilot, three to eight interventions were found per MR consultation. This finding is in line with the KNMP’s observation, as this organisation states that, on average, three to four PRPs are found in an MR and that this can increase to eight in vulnerable patients [1].

Our study further shows that an MR in Aruba can generate cost savings as well. This is in line with findings in the literature [1,37,38,39,40,41,42,43,44], although there are also sources that mention a finding of no significant cost savings [8,45]. This demonstrates that uncertainties remain about the cost-effectiveness of MR interventions in general. However, results from economic evaluations of MR interventions using STRIP as a support tool for multimorbid older adults (aged ≥ 65–70 years) with polypharmacy in Swiss primary care and in Swiss, Belgian, and Irish hospital settings did show that MRs have the potential to be cost-effective, since they can generate cost-savings and Quality-Adjusted Life Year (QALY) gains [37,46]. Especially in Aruba, MRs may be cost-effective, because treatment recommendations can take into account updated import prices of similar medicines, enabling more sustainable prescribing of medication. Moreover, the cost–benefit analysis of this study does not include the effects of an MR in terms of the prevention of drug-related problems and resulting hospitalisations. Therefore, looking at international publications considering these aspects, it is assumed that the cost savings are higher in practice than those identified in our study [47,48,49].

### 4.6. Future Steps in the Rollout of MRs in Aruba

Botica di Servicio hopes to demonstrate the added value of MRs in Aruba, and to show cost savings via a two-year extended pilot project. With that project, A.T. and A.W. from Botica di Servicio want to make the pharmacist and his/her tasks more visible in the Aruban healthcare chain. It is to be expected that it takes time to build a relationship of trust with patients so that they feel comfortable sharing information. Moreover, in the beginning, it will probably take time to explain the purpose of the MR consultation and what the remainder of the conversation will look like. This will certainly be the case for the members of the older population who are eligible for MRs due to polypharmacy.

It may also be that members of the older population are in great need of a conversation with a healthcare provider, which means that they also want to talk about general, non-medically related aspects, such as their family, grandchildren, particular situations, or current affairs. Taking time for this is important for building up a relationship with the patient. Knowledge about the family and surroundings of a patient is important for the good use of medicines. Meanwhile, the pharmacist must focus on the goal of the conversation.

During the implementation phase of the two-year pilot, it will be important that GPs, pharmacists, GP assistants, and pharmacy technicians collaborate intensively. Botica di Servicio wants to divide the tasks between pharmacists (1.5 h per MR), and pharmacy technicians (0.25 h per MR). Pharmacists will perform the analysis and conversations with the patients, and the pharmacy technicians will invite the patients to the MRs, in the meantime collecting some relevant information about the patient. To carry out MRs, both pharmacists and pharmacy technicians need to take training and courses about having motivational conversations with patients, and regarding the setup of an MR. There should be a focus on training about the themes of the MR, which are medications, adverse events, practice, and lifestyle. Furthermore, GPs and pharmacists should work closely together, with an open attitude towards regular consultation with each other, and see whether the structure of the MR is still ideal to maintain or should need adjustments. Subsequent to this, GPs and pharmacists can inform other healthcare providers in Aruba, such as psychologists, dietitians, physiotherapists, and medical specialists, about the existence of MRs for primary care. Overall, pharmacists need to continuously stay in contact with all stakeholders during the implementation phase. One tool that could be used for implementing MRs is the Consolidated Framework of Implementation Research (CFIR), which focuses on medication reviews [16].

### 4.7. Limitations and Strengths

This study contained several limitations. First, interviews with the community pharmacists and GPs were not carried out in a corresponding manner, as pharmacists were interviewed in a focus-group setting and GPs were interviewed one-to-one. Consequently, for example, pharmacists did not provide their opinions on the required patient selection process. However, the GPs provided a wide range of opinions on this subject, providing much advice that can be used for the implementation of MRs.

Second, during the interviews with GPs, the way in which questions were asked sometimes differed in each interview due to time constraints, which could have influenced the outcomes of the interviews. Nonetheless, the core of each interview was derived similarly as a standard approach, and a standardised survey protocol was employed.

A third point refers to the pilot MRs. M.L.C. and E.A.K. performed all MR consultations together, but the person who took the lead varied, which could have influenced the outcomes of the MR. On the other hand, different people can bring in different views on conducting MRs, offering more insights.

Furthermore, one limitation of the pilot MR is that the small sample size constrains the generalisability of the (cost–benefit analysis) findings. Moreover, the pilot MR only included male patients. Therefore, the needs of female patients may not be adequately reflected in the pilot research. The selection of patients was based on the patient’s ability to understand and speak the Dutch or English language and their capacity to participate in the pilot MR at short notice, i.e., within two weeks after actively being approached by the community pharmacy. Unfortunately, despite patients not being selected according to gender, this convenience sampling approach unconsciously led to the sole inclusion of male patients.

Another limitation concerns the included GPs. The GPs who were contacted by the pharmacists were also GPs with whom the relationship with the pharmacy was good. It cannot be ruled out that selection bias emerged, meaning that other non-participating GPs in Aruba may not perceive the added value of MRs, or that they consider today’s pharmaceutical care to be of sufficient quality.

Finally, during data collection and analysis, it might have been better to code all results, and write a codebook. This would have made parts of the data analysis more structural. In retrospect, this would have been more convenient, but no audio recordings had been made of the interviews, which means that transcripts could not be made and therefore, coding was more difficult. If coding had been done, quotes could have been added giving extra information and insights which would have been valuable for this study.

One strength was that the GP and community pharmacist study population was diverse, based on various nationalities and backgrounds, and with associated medical centres scattered across the island.

Another strength of the study was the many feedback moments between the authors while setting up and adjusting the MR protocol. Furthermore, this study included all target groups associated with an MR, namely community pharmacists, GPs, and patients, taking into account their views, expectations, experiences, and feedback. Altogether, our pilot study provided a comprehensive overview of the kind of MR design that is suitable for Aruban healthcare.

### 4.8. Future Research

Future research could provide more insights into the implementation of Aruban MRs regarding efficiency, workload, and effects on patients. For example, the question of how many drug-related problems can be covered by conducting MRs can be investigated. Another future research focus may include the economic evaluation of MRs in Aruba. This study only provided a crude estimate of the potential cost-savings, as the pilot MR was performed with a small patient sample and it did not include the costs associated with performing an MR, or the benefits of preventing drug-related problems and hospitalisations. This extensive cost–benefit knowledge could contribute to compensation arrangements for MRs in Aruba. Furthermore, the established MR protocol could be used in other countries as well, especially if these countries experience limited health literacy and extensive use of herbal products. When other countries use this format, researchers should critically evaluate which of its components would fit the specific country.

## 5. Conclusions

This study shows how MRs might be implemented on the island of Aruba, specifically taking into account the Aruban healthcare system and cultural themes. The included healthcare providers, both GPs and pharmacists, consider the main goal of MRs to be optimising the patient’s experience and treatment. Patient selection and follow-up for MRs should be performed in collaboration between GPs and pharmacists. Patients who use at least five different types of chronic medication or struggle with limited health literacy should be selected for MRs, regardless of age. Regarding the relevant (cultural) themes for MRs in Aruba, healthcare providers should be aware of lifestyle factors, limited health literacy, the use of herbal products, patients’ hierarchical view of GPs, and potential barriers of high workload and miscommunication between healthcare providers. In the future, healthcare providers should make clear (scheduled) agreements together to implement MRs in Aruba. This study provides a format for performing MRs in Aruba, but other countries with similar characteristics could also make use of (an adapted version of) the established format. Specifically, Caribbean islands that are part of the Kingdom of the Netherlands may directly benefit from the findings in this study, due to cultural similarities between these islands. Future research can either provide formats fitting other countries or continue to improve the established format for Aruba by considering the experiences of each stakeholder on a more in-depth basis. The cost-saving potential of MRs in Aruba is outlined, but more extensive research is needed to generalise the findings and make cost-effectiveness claims. In general, this pharmacy practice study offers the first version of an MR protocol specifically fitting the Aruban healthcare system and culture, underlining the importance of multidisciplinary collaboration.

## Figures and Tables

**Table 1:** Description of the study participants: general practitioners, pharmacists, and patients.

Study Population	Participant	Location on Island	Gender	Country of Education	Origin	Length of Residence in Aruba as a Healthcare Provider	Experience with Conducting an MR
General Practitioners (*n* = 8)	GP 1 *	North	Female	NL	Aruban	1 year	No
	GP 2	North	Female	NL	Dutch	1 year	Yes
	GP 3	North	Male	NL	Spanish (Latin American)	12 years	No
	GP 4 *	North	Female	NL	Dutch	4 months	Yes
	GP 5	North	Female	Belgium	Belgian	5 years	No
	GP 6	Middle	Female	NL	Aruban	18 years	No
	GP 7	Middle	Female	Suriname	Surinam	3 years	Yes
	GP 8	South	Female	NL	Aruban	10 years	No
Pharmacists (*n* = 5)	Pharmacist 1	North	Female	NL	Dutch	5 years	Yes
	Pharmacist 2 *	North	Female	NL	Aruban	6 years	Yes
	Pharmacist 3	North	Male	NL	Aruban	20 years	No
	Pharmacist 4	Middle	Female	NL	Dutch	17 years	No
	Pharmacist 5	South	Male	NL	Dutch	22 years	No
Patients (*n* = 4)	Participant	Age	Gender	Language	Origin	Number of Medicines in Use	Number of Comorbidities
	Patient 1	61 years	Male	Spanish and Papiamento	Spanish (Latin-American)	5	3
	Patient 2	72 years	Male	Dutch	Dutch	5	4
	Patient 3	73 years	Male	English	Scottish	10	3
	Patient 4	78 years	Male	Dutch and Papiamento	Aruban	9	3

* These healthcare professionals were involved in the pilot MR.

**Table 2:** Overview of data processing of the interviews with GPs, pharmacists, and the pharmacy owner. Results were coded or summarised, as indicated.

Subject Interview	Data Analysis Interviews
Patient selection	Coding: age and number of medicines
Aruba, local aspects	Coding: healthcare, conditions, herbal and self-care products, behaviour/culture, adherence
Protocol MR (only first version)	Points of interest summarised, divided into ‘pharmaceutical history’ and ‘carrying out an MR’
Delegation of tasks	Coding: patient selection and follow-up

**Table 3:** Overview of data processing for the pilot MR. The table indicates how the data processing was carried out.

Subject Pilot	Data Analysis Pilot
Patients’ reactions	Quotes
Intervention, MRs	Quantitative measurements of interventions in the areas of ‘medication’, ‘practice’, ‘complaints’, and ‘lifestyle’.
Elaboration, MRs	Summarised points needing attention for ‘implementation of MR’.

**Table 4:** Overview of important points for carrying out an MR as a whole, according to the interviewed healthcare providers.

Area	Results
Inviting patients	Invite the patient via telephone or WhatsApp (mobile messaging service). Have the patient come for an MR once every three years.
	Invite five to six patients every two months.
Anamnesis	Keep the conversation with patients as concise as possible.
	Mention that everything will be discussed with the GP. Mention professional secrecy. Clearly explain changes in the medication list.
Drawing up a treatment plan	Use the BAAN form. *
	Send the BAAN form to the GP a week before the GP–pharmacist meeting. Discuss all patients at once with the GP upon completion of the MR consultations.
Follow-up	Pharmacists: GP should carry out follow-up; GPs: collaboration between pharmacist and GP
	Collect patient’s old medication boxes during follow-up or earlier. Ask about the patient’s views on previous concerns/beliefs. Have the patient come to the pharmacy, to analyse non-verbal communication. GPs: let the patient make a follow-up appointment themselves.

* A summary form containing patient details; dates for the MR planning; additions, removals, and adjustments from the medication list; questions to ask the GP; and an overview of the history of the patient’s conditions, with associated laboratory values.

**Table 5:** Overview of important points for carrying out the pharmacotherapeutic anamnesis, according to the interviewed healthcare providers.

Area	Results
Medication	Assess understanding of the reason for using the medication.
	Ask about herbal/over-the-counter remedies.
Complaints	Ask about stiffness/pain in joints, psychological complaints, and forgetfulness. Use smiley scales for limited health literacy. Assess understanding of labels by having the patient read the label and explain the interpretation.
	Be careful not to mention all adverse effects in the list. *
Practice	Ask how the medication is stored by the patient. Request instructions for the use of medication (e.g., inhalation instructions, creams, and eye drops) Ask about concerns, beliefs, and practical problems related to non-adherence. Refer the patient to videos from Kijksluiter ** or YouTube *** where necessary.
Lifestyle	Ask the question ‘What are stumbling blocks/problems in your life?’. Pharmacist should refer to GP when a patient experiences lifestyle problems. Mention wine and beer when asking about how much alcohol the patient drinks. Ask what someone eats during the day. Verify in a pilot whether lifestyle suits an MR.

* While setting up MRs in Aruba, a preliminary document was made as a tool for carrying out the pharmacotherapeutic anamnesis, among others. This document contains a list of frequently occurring medication adverse events. ** www.kijksluiter.nl (accessed on 4 July 2024), *** www.youtube.com (accessed on 4 July 2024).

**Table 6:** Overview of the calculations performed in determining the cost savings of MR1 as a result of (proposed) changes in medication policy. The patient’s age at the time of the MR consultation (June 2023) was 72 years, meaning that a remaining life duration of five years was taken into account (based on the current life expectation of the Aruban population).

Changes in Medication Policy due to the MR	Type of Medication	Total Import Price of the Particular Medication Package(s), Corresponding to Three Months of Use (AWG)	Expected Number of Dispensing Moments	Overall Savings and Costs (AWG)
Stop	Clopidogrel 75 mg tablet	37.70	20	−754
	Coveram 5 mg tablet	141.05	20	−2821
	Atorvastatin 40 mg tablet	51.20	20	−1024
	Ascal 100 mg tablet	30.50	20	−610
Start	Acetylsalicylic acid 80 mg tablet	21.50	20	+430
	Rosuvastatin 5 mg tablet	26.00	20	+520
	Perindopril 4 mg tablet	27.58	20	+551.60
Total (AWG)				−3707.40

**Table 7:** Overview of the results of the pilot, with comments on several areas. The comments about the anamnesis are divided into four areas: ‘medication’, ‘practice’, ‘complaints’, and ‘lifestyle’.

Subject	Results
MR in general	An MR must be adapted to a patient’s level of understanding. Do not explain laboratory values to a patient. Anamnesis with the patient took 1 to 1.5 h. An interpreter was needed once (Spanish/Papiamento-Dutch) None of the patients had medicine boxes with them, even when they were asked to bring them to the anamnesis.
Patient selection	GPs found it difficult to select patients.
Approaching the GP (practice before anamnesis)	Some important values were missing in the received episode list and laboratory values. Laboratory values are displayed with different units in varying GP practices.
Approaching a patient during anamnesis	Questions about medication satisfaction, adverse events, and times of administration were not answered properly on the telephone. The above also emerged during the anamnesis. One patient may not have been honest about his medication adherence
Anamnesis (medication)	Three patients had little or no knowledge about the reason why they took certain types of medicines. None of the patients answered questions about taking over-the-counter or herbal remedies.
Anamnesis (practice)	Two patients taking eye droplets needed instructions to apply the droplets properly. Patients seemed to understand the questions about the use in practice. Two patients stored their medication in the wrong place.
Anamnesis (complaints)	One patient may have had muscle complaints due to simvastatin, which prevented him from moving during the day. Three patients suffered from pain complaints, for which they were advised to try paracetamol. Three patients only used paracetamol for headache and fever. Two patients were asked to read medication labels. Both patients performed this successfully.
Anamnesis (lifestyle)	One patient was addicted to smoking and wanted guidance from the GP practice. Two patients did not want to make any lifestyle changes. All patients understood the lifestyle card.
Evaluation with the GP	Before sending the BAAN form to the GP, it turned out to be useful to contact the purchasing pharmacist about the prices of medicines, as this enabled recommendations to be made to the GP about similar/equivalent medications that were economically more favourable (lower-priced).

## Data Availability

The datasets for this manuscript are not publicly available because of privacy measures. Requests to access the datasets should be directed to the second author and will be granted on reasonable request.

## Supplementary Materials

The following supporting information can be downloaded at: https://www.mdpi.com/article/10.3390/pharmacy12040108/s1, Overview S1: First version of a protocol for conducting MRs in Aruba; Overview S2: Surveys for Aruban GPs and community pharmacists; Overview S3: Survey for Aruban patients participating in the pilot study; Overview S4: Three subsequent versions of the protocol for conducting MRs in Aruba, being adjusted versions of Overview S1 based on feedback received during the pilot study.
