# Supplementary material for: Developing Medication Reviews to Improve the Aruban Healthcare System: A Mixed-Methods Pilot Study
Source: Pharmacy (Basel). 2024 Jul 12;12(4):108. doi: 10.3390/pharmacy12040108 (PMC11270182; doi:10.3390/pharmacy12040108)
Supplement: Supplementary file 1 [file pharmacy-12-00108-s001.zip › Supplementary Materials Pharmacy-3059560_final.pdf]

Supplementary Materials

Developing Medication Reviews to Improve the Aruban Healthcare System: A Mixed-Methods Pilot Study

Minke L. Copinga [1,†], Ellen A. Kok [1,†], Anke J. J. van Dam [2], Anoeska Wever [3], Adrienne Tromp [3,*] and Herman J. Woerdenbag [4,*]

1 Pharmacy Master Programme, School of Science and Engineering, University of Groningen, Antonius Deusinglaan 1, 9713 AV Groningen, The Netherlands; minkelouisecopinga@gmail.com (M.L.C.); ellen.kokx@hotmail.com (E.A.K.).
2 Pharos, Expertise Center on Health Disparities; Arthur van Schendelstraat 600, 3511 MJ Utrecht, The Netherlands; a.vandam@pharos.nl (A.J.J.V.D.)
3 Botica di Servicio, Caya Punta Brabo 17, Oranjestad, Aruba; awever@boticadiservicio.com (A.W.); adrienne@boticadiservicio.com (A.T.)
4 Department of Pharmaceutical Technology and Biopharmacy, Groningen Research Institute of Pharmacy (GRIP), University of Groningen, Antonius Deusinglaan 1, 9713 AV Groningen, The Netherlands; h.j.woerdenbag@rug.nl (H.J.W.)
* Correspondence:
University of Groningen: h.j.woerdenbag@rug.nl; (H.J.W.); Tel.: +31-631921365
Botica di Servicio: adrienne@boticadiservicio.com; (A.T.); Tel.: +297-6408443
† These authors contributed equally to this work.

Table of Contents

Overview S1. First version of a protocol for conducting MRs in Aruba.

Overview S2. Surveys for Aruban GPs and community pharmacists.

Overview S3. Survey for Aruban patients participating in the pilot study.

Overview S4. Three subsequent versions of the protocol for conducting MRs in Aruba, being adjusted versions of Overview S1 based on feedback received during the pilot study.

Overview S1. First version of a protocol for conducting MRs in Aruba

(Translation of the original Dutch document.)

1. Preparation

1.1. Patient data

Request the following patient information from the GP: episode list, active medication, stopped medication (past year), lab values, height, and weight. Remove the patient's sensitivities and contraindications from the AIS*. [*AIS = Apotheek Informatie Systeem (Pharmacy Information System)]

Copy all the data here:

1.2. Sample phone call to introduce a medication review

Preparation: during the conversation, keep the medication list next to you with the dosing frequencies and dosages.

Introduce yourself: 'I am (name), pharmacist of Botica di Servicio <name of pharmacy>.'

Ask if you're calling at the right time.

Ask if you are calling the right person (→ name, street, date of birth, AZV** number, etc.)

[**AZV = Algemene Ziektekosten Verzekering (National Ordinance General Health Insurance of Aruba)]

Explain: 'Together with my GP, I checked whether there are patients who take a lot of medicines. It is important that we check with this patient how the medication is going now. We will then check whether your illnesses are being treated properly and whether some medication needs to be added or can be removed. We would also like to see whether you, as a patient, also experience problems or wishes when it comes to medication use. This allows us to better tailor your medication use to your situation. Would you like to take a look at your medication with me?'

If the patient says no:

'That's good. If you would like to have a conversation in the future, please let us know. Then I wish you a nice day!'

If the patient says yes:

'How nice that you want to have a conversation about your medication. I will then ask a few small questions so that I can properly prepare for my conversation with you. After that, I will arrange a date and location with you. Is that okay with you?'

Other, namely:

Satisfaction about medication use	Does it have an effect? Yes/No<br>Can medication use be incorporated into your daily life? Yes/No		
Intake moments: is this going well?	When is it difficult to take the medication?		
Type of adverse events	Never	Sometimes	Often
Dizziness			
Drowsiness			
Fatigue/sleep problems			
Shortness of breath			
Muscle pain/weakness			
Bruises/bleeding			
Dry skin/itching			
Diarrhea/constipation/stomach complaints			
Dry mouth			
Problems with urinating			
Sexual problems			
Other adverse events:			

Say: 'Thank you for all the information. For the interview, it is useful if you bring all your medicines with you. This includes boxes of medicines, but also creams, puffs, painkillers and herbal remedies. Think of paracetamol, St. John's wort, vitamins and multivitamins and other products that you are thinking of. This gives us a complete picture of what you are using and we can advise on it.'

'Then I would like to schedule a date with you now. When would you be able to meet?'
- Date:
- Time:
- Location:

Tell the patient that there will be another email with the date, time and location and close the conversation.

Analysis

Overtreatment	
Undertreatment	
Incorrect dosage	
Dual medication	
Off-label use	
Incorrect/impractical forms of administration	
Interactions	
Contraindications	
Avoidable adverse events	
Ineffective medication	
Non-adherence	

1.3. Email to the patient after the phone call

Dear Mr/Ms/Mrs <name>,

I am <name>, pharmacist at Botica di Servicio <name of pharmacy>. We had a conversation about a medication review. In this conversation, you mentioned that you would like us to carry out this medication review. The medication review consists of a conversation with you about your medications. During this meeting, we would like to discuss your medication and wishes with you. That way, we hope to find the problems that you are experiencing. We will work together to find solutions to this.

The appointment for the medication review:

Date: XX

Time: XX

Location: XX

If the conversation takes place in the pharmacy: Don't forget to take your medication with you to the pharmacy. Think of pills, creams, puffs, and plasters. We would like to ask you to also bring products that you can buy in the drugstore without a prescription from your doctor. This helps us get a good idea of your medications.

See you soon!

Sincerely,

<name>, pharmacist at Botica di Servicio <name of pharmacy>

2. Pharmacotherapeutic anamnesis

2.1. Patient records

Name	
Age	
Address	
AZV-number	
Medical specialists and other doctors who treat you	

2.2. Medication use

Type of medication	Reason for patient use + method of use (dosage, frequency)

Herbal remedies/self-care remedies?
E.g., paracetamol, St. John's wort, creams, fish oil, multivitamins, other vitamins.

Type of medication	Dosage	Frequency	Obtained from where?

Patient's goals:
What does the patient want to change/what are their goals for the conversation (if the patient doesn't understand the question, explain to the patient: think of less pain, fewer pills, more mobility).

Patient-specific questions:

2.3. Complaints

Does medication help?<br>Yes/no<br><br>Explanation:

Pain: Can you indicate how much pain you are currently in by looking at the emotions below?

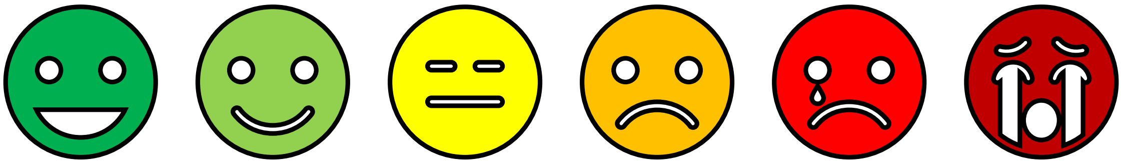

No Mild Moderate Severe Very Severe Intolerable

0 1 2 3 4 5 6 7 8 9 10

Where is the pain, when does it occur and what does that pain feel like?

You have already indicated which adverse effects you have due to your medication. How bad do you assess the worst adverse effects?

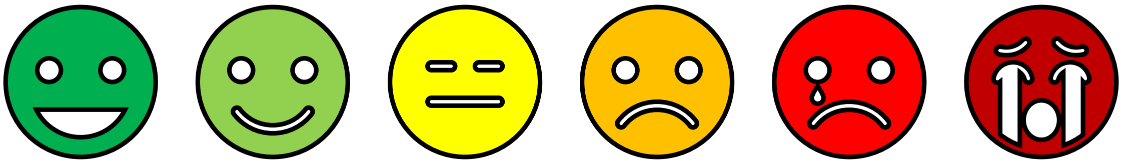

None Mild Moderate Severe Very severe Intolerable

0 1 2 3 4 5 6 7 8 9 10

If you have multiple adverse events, which events are the worst?
1.
2.
3.

How often do you suffer from the adverse events?

Type of adverse event	Indicate how often, e.g. daily, every now and then, before bed, at night, etc.
Dizziness	
Drowsiness	
Fatigue/sleep problems	
Shortness of breath	
Muscle pain/weakness	
Bruises/bleeding	
Dry skin/itching	
Diarrhea/constipation/stomach complaints	
Dry mouth	
Problems urinating	
Sexual problems	
Otherwise	

What grade would you give your life right now?

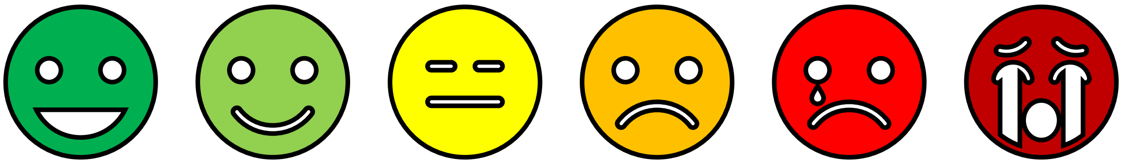

No Mild Moderate Severe Very Severe Intolerable

0 1 2 3 4 5 6 7 8 9 10 Mild

Explanation:

Mental health: How does the patient feel? Major events? Feeling comfortable or uncomfortable, anxiety, panic, feeling depressed? Anxiety can manifest itself in: high heart rate, dry mouth, shortness of breath, tightness.[1]

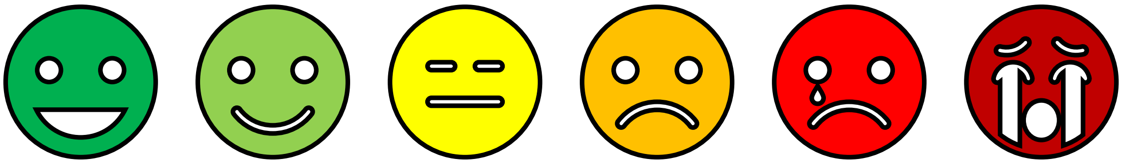

No Mild Moderate Severe Very Severe Intolerable

0 1 2 3 4 5 6 7 8 9 10 Mild

Explanation:

Patient's own interpretation:

Memory/cognition: memory problems? Think of forgetfulness, not knowing what day it is, not being able to find words, not being able to solve problems, or getting lost [2,3]

Yes/no

Explanation:

Morning stiffness/pain in joints

Yes/no

Explanation:

Label readable

Explanation: show label to patient: 'Can you read this label and explain how to use this medicine?'

Yes/no

Explanation:

---

[1]Brain Foundation. Mental health. Available from: https://www.hersenstichting.nl/mentale-gezondheid/

[2]Brain foundation. Functions of your brain. Available at: https://www.hersenstichting.nl/de-hersenen/werking-van-de-hersenen/functies/

[3]Rijnstate. Cognitive impairment. Available from: https://www.rijnstate.nl/aandoening-en-behandeling/cognitieve-stoornissen/

2.4. Practice

Daily use:
What times of the day do you take your medications?
Is it always possible to take the medication at the right time? Yes/no
When do you find it difficult to take your medication?
Do you ever take someone else's medication?

Storing medicines:
Where do you store your medicines at home?

Practical problems:
Think of difficulty opening packaging, difficulty opening blisters, difficulty swallowing, difficulty using puffs, etc.

Asthma/COPD inhalation instruction:
Can you show us how you use your pufs?
(Give inhalation instruction in the case of wrong execution)

Use of creams:
Can you explain how you use your creams?

2.5. Lifestyle

Put a maximum of 3 crosses at the points (in the figure below) you would like to work on.

Translation Dutch-English in the figure below:
Upper row, left to right: movement, food, alcohol, smoking, weight,
Middle row, left to right: medication, family, money, work, stress,
Lower row left to right: social environment, blood pressure, daily pattern, sleeping, drinking

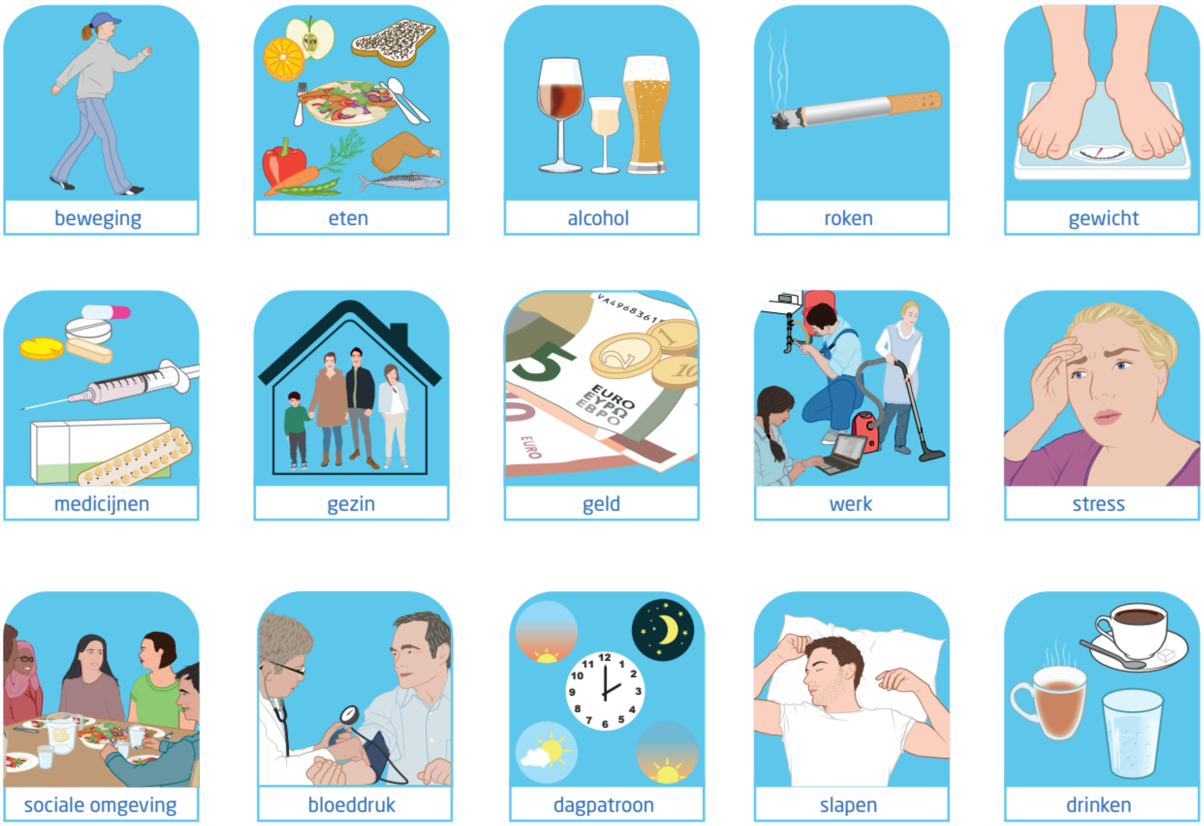

Number of glasses of water/tea per day:
Daily dairy intake:
Number of glasses of alcohol per day:

Smoking yes/no:	How much per day:
Drug use yes/no:	How much:
How long per day exercise:	hours
How long per day outside:	hours

Talk to the patient: as a pharmacist, I can help with lifestyle.

What would you like help with?

Would you like a more extensive conversation about lifestyle?

Can I share this information with your GP? Yes/no

2.6. Lifestyle consultation (separate from MR):

Draw up a plan together with the patient to work on the most important lifestyle point (this is during a separate lifestyle consultation). Translation Dutch-English: questions about daily functioning, bodily functions, mental well-being, meaning, quality of life, participation.

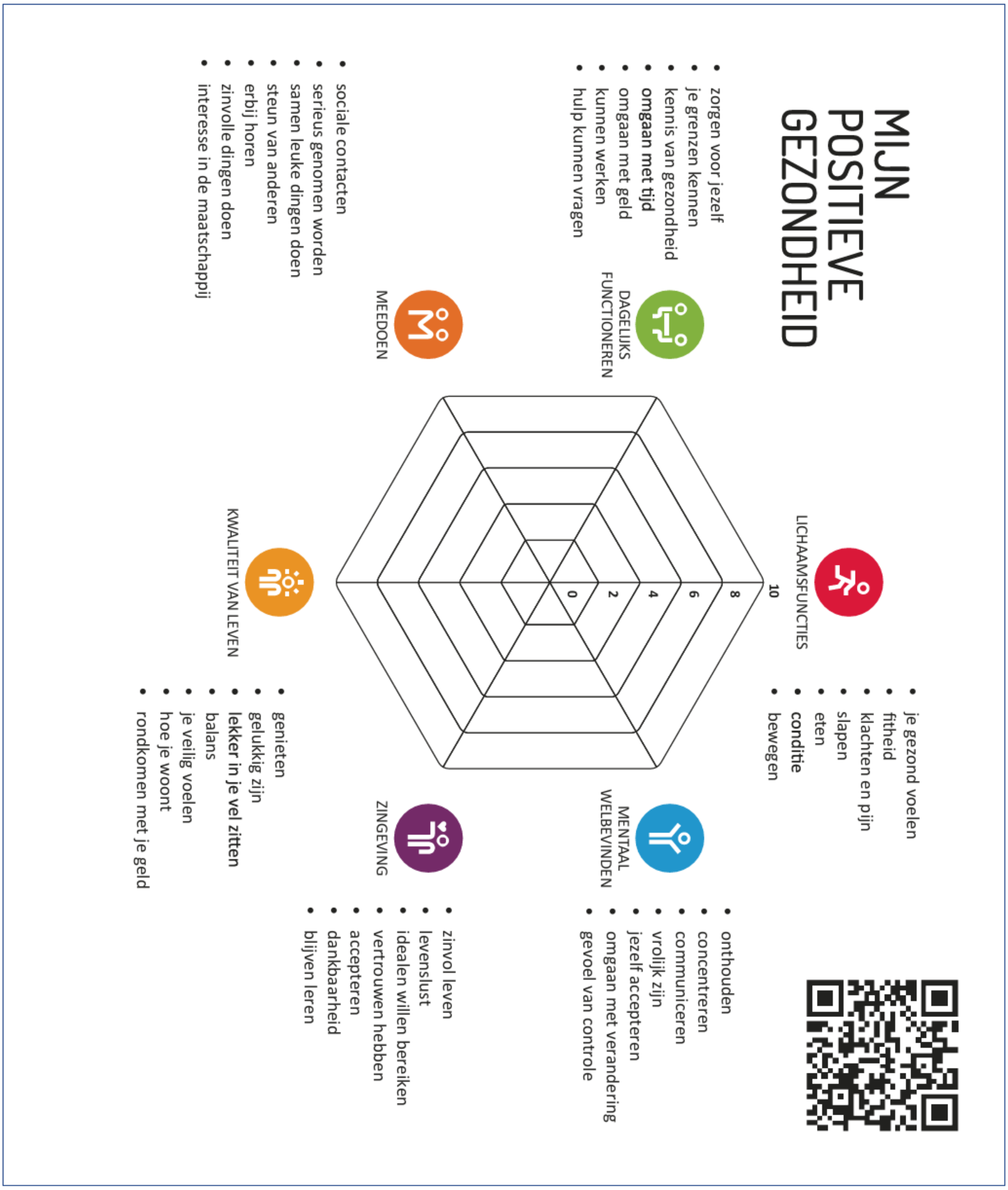

3. Pharmacotherapeutic analysis

3.1 Appointments, discussed later with the GP

Send the following points to the GP by email, one week before the appointment with the GP:

Add	Remove	Different	Inquire

Action points from 'Complaints' (see pharmacotherapeutic anamnesis):

Action points from 'Practice' (see pharmacotherapeutic anamnesis):

Action points from 'Lifestyle' (see pharmacotherapeutic anamnesis):

Summary anamnesis:

<u>Comments</u>

Medication + dosage	Episode	Use	Action needed? Yes/no	Remarks

Relevant lab values (copy from preparation):

Contraindications and sensitivities (copy from preparation):

4. Drawing up a treatment plan based on the result of the dialogue between doctor and pharmacist

4.1. Treatment Plan

Action point	Execution period	Which healthcare provider does follow-up	Date of follow-up

4.2. Phone call with the patient after the appointment with the GP

Discuss all agreements that have been made with the doctor. Discuss with the patient whether he or she agrees with the agreed points. If the patient agrees, the treatment plan of 4.1 is fixed. If the patient does not yet agree or if an action point is adjusted, it may be necessary to talk/email the GP again.

5. Follow-up

Call the patient on the agreed date (see 4. Drawing up a treatment plan) and record the patient's experiences.

Action point	Results and experiences according to the patient

Overview S2. Surveys for Aruban GPs and community pharmacists.

A. Form for GPs - Implementation of medication reviews in Aruba (T=0)

Circle the answer that applies the most. For questions 7, 10, and 11, you have to fill in your own answer.

1. Have you heard of the term "medication review" before?
   a. Yes
   b. No

2. I can explain to a patient what a medication review entails.
   Not – Little – Moderate – Neutral – Satisfactory – Good – Excellent

3. General practitioners in Aruba need (further) training about (the implementation of) medication reviews and about what they can do for patient care.
   a. Agree
   b. Disagree

4. Medication reviews can be of added value in primary care in Aruba.
   Strongly Disagree – Disagree – Neutral – Agree – Strongly Agree - No opinion

5. Would you be willing to carry out medication reviews in Aruba in collaboration with Botica di Servicio in a pilot project?
   Very Unlikely – Unlikely – Neutral – Likely – Very Likely – No Opinion

6. Is it negotiable for you to take advice on possible adjustments to medication (or therapy) from the pharmacist?
   Never – Sometimes – Often – Always

7. What do you think is the degree of ultimate responsibility per party involved in a medication review? Express this in a percentage.[1]
   General practitioner %............/100%
   Pharmacist %............/100%
   Patient %............/100%

[1] Elyan, J. et al. Understanding the potential for pharmacy expertise in palliative care: the value of stakeholder engagement in a theoretically driven mapping process for research. *Pharmacy* **2021**, **9**, 192.

8. What do you think is the main purpose of a medication review?[2]
   a. Best clinical outcome for the patient
   b. Cost-effectiveness
   c. Optimising the patient experience – aligning preferences and needs to promote adherence (concordance)

9. I expect .... will be an obstacle to the conduct of medication reviews in Aruba.[3,4]
   a. Miscommunication between GP and pharmacist
      Strongly disagree - disagree - neutral - agree - strongly agree

   b. Variability in the course of the disease (e.g. temporary symptoms)
      Strongly disagree - disagree - neutral - agree - strongly agree

   c. Variability in patient wishes
      Strongly disagree - disagree - neutral - agree - strongly agree

   d. Hierarchy in healthcare
      Strongly disagree - disagree - neutral - agree - strongly agree

   e. Limited time / high workload
      Strongly disagree - disagree - neutral - agree - strongly agree

   f. Unclear responsibility
      Strongly disagree - disagree - neutral - agree - strongly agree

   g. Content complexity of medication reviews
      Strongly disagree - disagree - neutral - agree - strongly agree

10. Can you indicate what you think will be the most hindering factor in question 9 from options a to g, and can you possibly give an explanation?

11. Optional: space for additional comments about (setting up) medication reviews in Aruba.

---

[2] Smith, I.; Hicks, C.; McGovern, T. Adapting lean methods to facilitate stakeholder engagement and co-design in healthcare. *BMJ* **2020**, *368*, m35.
[3] Elyan, J. et al. Understanding the potential for pharmacy expertise in palliative care: the value of stakeholder engagement in a theoretically driven mapping process for research. *Pharmacy* **2021**, **9**, 192.
[4] Jahangirian, M. et al. Causal factors of low stakeholder engagement: a survey of expert opinions in the context of healthcare simulation projects. *Simulation: Transactions of the Society for Modeling and Simulation International* **2015**, *91*, 511–526.

B. Form for GPs - Implementation of medication reviews in Aruba (T=1)

Circle the answer that applies the most. For questions 6, 9, and 10, you have to fill in an answer yourself.

1. I can explain to a patient what a medication review entails.
   Not – Little – Moderate – Neutral – Satisfactory – Good – Excellent

2. General practitioners in Aruba need (further) training about (the implementation of) medication reviews and about what they can do for patient care.
   a. Agree
   b. Disagree

3. Medication reviews can be of added value in primary care in Aruba.
   Strongly Disagree – Disagree – Neutral – Agree – Strongly Agree - No opinion

4. Would you be willing to carry out medication reviews in Aruba in collaboration with Botica di Servicio in a pilot project?
   Very Unlikely – Unlikely – Neutral – Likely – Very Likely – No Opinion

5. Is it negotiable for you to take advice on possible adjustments to medication (or therapy) from the pharmacist?
   Never – Sometimes – Often – Always

6. What do you think is the degree of ultimate responsibility per party involved in a medication review? Express this in a percentage.[5]
   General practitioner %............/100%
   Pharmacist %............/100%
   Patient %............/100%

7. What do you think is the main purpose of a medication review?[6]
   a. Best clinical outcome for the patient
   b. Cost-effectiveness
   c. Optimising the patient experience – aligning preferences and needs to promote adherence (concordance)

[5] Elyan, J. et al. Understanding the potential for pharmacy expertise in palliative care: the value of stakeholder engagement in a theoretically driven mapping process for research. *Pharmacy* **2021**, *9*, 192.
[6] Smith, I.; Hicks, C.; McGovern, T. Adapting lean methods to facilitate stakeholder engagement and co-design in healthcare. *BMJ* **2020**, *368*, m35.

8. I expect .... will be an obstacle to the conduct of medication reviews in Aruba.[7,8]
   a. Miscommunication between GP and pharmacist
      Strongly disagree - disagree - neutral - agree - strongly agree

   b. Variability in the course of the disease (e.g. temporary symptoms)
      Strongly disagree - disagree - neutral - agree - strongly agree

   c. Variability in patient wishes
      Strongly disagree - disagree - neutral - agree - strongly agree

   d. Hierarchy in healthcare
      Strongly disagree - disagree - neutral - agree - strongly agree

   e. Limited time / high workload
      Strongly disagree - disagree - neutral - agree - strongly agree

   f. Unclear responsibility
      Strongly disagree - disagree - neutral - agree - strongly agree

   g. Content complexity of medication reviews
      Strongly disagree - disagree - neutral - agree - strongly agree

9. Can you indicate what you think will be the most impeding factor in question 8 from options a to g, and can you possibly give an explanation?

10. Optional: space for additional comments about (setting up) medication reviews in Aruba.

---

[7] Elyan, J. et al. Understanding the potential for pharmacy expertise in palliative care: the value of stakeholder engagement in a theoretically driven mapping process for research. *Pharmacy* **2021**, **9**, 192.
[8] Jahangirian, M. et al. Causal factors of low stakeholder engagement: a survey of expert opinions in the context of healthcare simulation projects. *Simulation: Transactions of the Society for Modeling and Simulation International* **2015**, *91*, 511–526.

C. Form for community pharmacists - Implementation of medication reviews in Aruba (T=0)

Circle the answer that applies the most. For questions 7, 10, and 11, you have to fill in an answer yourself.

1. Have you ever conducted a medication review in primary care in the Netherlands?
   a. Yes, about ....
   b. No

2. I can explain to a patient what a medication review entails.
   Not – Little – Moderate – Neutral – Satisfactory – Good – Excellent

3. I am currently .... competent to conduct a medication review in collaboration with the GP in primary care in Aruba using a template.
   Not – Little – Neutral – Satisfactory – Excellent

4. Community pharmacists in Aruba need (further) training about (the implementation of) medication reviews and about what they can do for patient care.
   a. Agree
   b. Disagree

5. Medication reviews can be of added value in primary care in Aruba.
   Strongly Disagree – Disagree – Neutral – Agree – Strongly Agree - No opinion

6. Do you think it is achievable for you to convey advice on possible adjustments to medication (or therapy) to the GP in such a way that he considers and/or adopts it?
   Never – Sometimes – Often – Always

7. What do you think is the degree of ultimate responsibility per party involved in a medication review? Express this in a percentage.[9]
   General practitioner %............/100%
   Pharmacist %............/100%
   Patient %............/100%

---

[9] Elyan, J. et al. Understanding the potential for pharmacy expertise in palliative care: the value of stakeholder engagement in a theoretically driven mapping process for research. *Pharmacy* **2021**, *9*, 192.

8. What do you think is the main purpose of a medication review?[10]
   a. Best clinical outcome for the patient
   b. Cost-effectiveness
   c. Optimising the patient experience – aligning preferences and needs to promote adherence (concordance)

9. I expect .... will be an obstacle to the conduct of medication reviews in Aruba.[11,12]
   a. Miscommunication between GP and pharmacist
      Strongly disagree - disagree - neutral - agree - strongly agree

   b. Variability in the course of the disease (e.g. temporary symptoms)
      Strongly disagree - disagree - neutral - agree - strongly agree

   c. Variability in patient wishes
      Strongly disagree - disagree - neutral - agree - strongly agree

   d. Hierarchy in healthcare
      Strongly disagree - disagree - neutral - agree - strongly agree

   e. Limited time / high workload
      Strongly disagree - disagree - neutral - agree - strongly agree

   f. Unclear responsibility
      Strongly disagree - disagree - neutral - agree - strongly agree

   g. Content complexity of medication reviews
      Strongly disagree - disagree - neutral - agree - strongly agree

10. Can you indicate what you think will be the most impeding factor in question 9 from options a to g, and can you possibly give an explanation?

11. Optional: space for additional comments about (setting up) medication reviews in Aruba.

---

[10] Smith, I.; Hicks, C.; McGovern, T. Adapting lean methods to facilitate stakeholder engagement and co-design in healthcare. *BMJ* **2020**, *368*, m35.
[11] Elyan, J. et al. Understanding the potential for pharmacy expertise in palliative care: the value of stakeholder engagement in a theoretically driven mapping process for research. *Pharmacy* **2021**, **9**, 192.
[12] Jahangirian, M. et al. Causal factors of low stakeholder engagement: a survey of expert opinions in the context of healthcare simulation projects. *Simulation: Transactions of the Society for Modeling and Simulation International* **2015**, *91*, 511–526.

D. Form for community pharmacists - Implementation of medication reviews in Aruba (T=1)

Circle the answer that applies the most. For questions 6, 9, and 10, you have to fill in an answer yourself.

1. I can explain to a patient what a medication review entails.
   Not – Little – Moderate – Neutral – Satisfactory – Good – Excellent

2. I am currently .... competent to conduct a medication review in collaboration with the GP in primary care in Aruba using a template.
   Not – Little – Neutral – Satisfactory – Excellent

3. Community pharmacists in Aruba need (further) training about (the implementation of) medication reviews and about what they can do for patient care.
   a. Agree
   b. Disagree

4. Medication reviews can be of added value in primary care in Aruba.
   Strongly Disagree – Disagree – Neutral – Agree – Strongly Agree - No opinion

5. Do you think it is achievable for you to convey advice on possible adjustments to medication (or therapy) to the GP in such a way that he considers and/or adopts it?
   Never – Sometimes – Often – Always

6. What do you think is the degree of ultimate responsibility per party involved in a medication review? Express this in a percentage.[13]
   General practitioner %............/100%
   Pharmacist %............/100%
   Patient %............/100%

7. What do you think is the main purpose of a medication review?[14]
   a. Best clinical outcome for the patient
   b. Cost-effectiveness
   c. Optimising the patient experience – aligning preferences and needs to promote adherence (concordance)

---

[13] Elyan, J. et al. Understanding the potential for pharmacy expertise in palliative care: the value of stakeholder engagement in a theoretically driven mapping process for research. *Pharmacy* **2021**, *9*, 192.
[14] Smith, I.; Hicks, C.; McGovern, T. Adapting lean methods to facilitate stakeholder engagement and co-design in healthcare. *BMJ* **2020**, *368*, m35.

8. I expect .... will be an obstacle to the conduct of medication reviews in Aruba.[15,16]
    a. Miscommunication between GP and pharmacist
        Strongly disagree - disagree - neutral - agree - strongly agree

    b. Variability in the course of the disease (e.g. temporary symptoms)
        Strongly disagree - disagree - neutral - agree - strongly agree

    c. Variability in patient wishes
        Strongly disagree - disagree - neutral - agree - strongly agree

    d. Hierarchy in healthcare
        Strongly disagree - disagree - neutral - agree - strongly agree

    e. Limited time / high workload
        Strongly disagree - disagree - neutral - agree - strongly agree

    f. Unclear responsibility
        Strongly disagree - disagree - neutral - agree - strongly agree

    g. Content complexity of medication reviews
        Strongly disagree - disagree - neutral - agree - strongly agree

9. Can you indicate what you think will be the most impeding factor in question 8 from options a to g, and can you possibly give an explanation?

10. Optional: space for additional comments about (setting up) medication reviews in Aruba.

[15] Elyan, J. et al. Understanding the potential for pharmacy expertise in palliative care: the value of stakeholder engagement in a theoretically driven mapping process for research. *Pharmacy* **2021**, **9**, 192.
[16] Jahangirian, M. et al. Causal factors of low stakeholder engagement: a survey of expert opinions in the context of healthcare simulation projects. *Simulation: Transactions of the Society for Modeling and Simulation International* **2015**, *91*, 511–526.

Overview S3. Survey for Aruban patients participating in the pilot study.

(This form was available in Dutch and English, but only the English version is shown below)

Client evaluation form based on the Generic Short Patient Experiences Questionnaire (GS-PEQ)[17]

1. The pharmacist spoke to me in a way that was easy to understand.

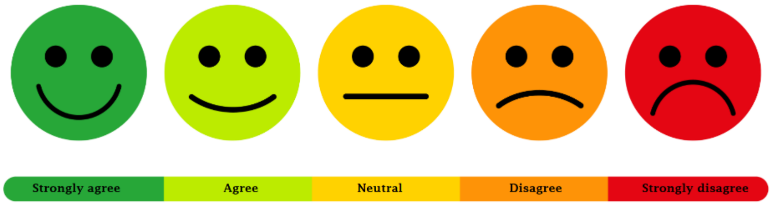

2. I have confidence in the pharmacist's knowledge.

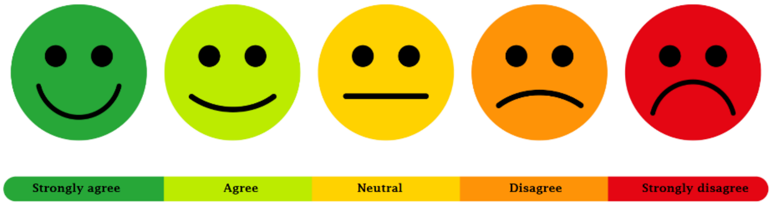

3. I received sufficient information about my drug treatment.

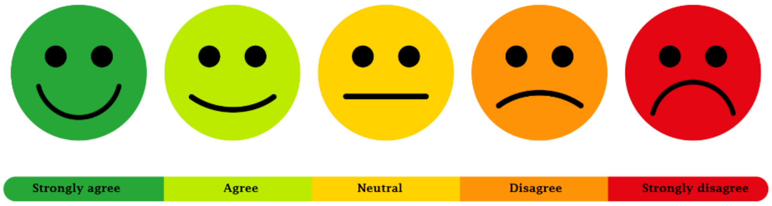

4. My personal situation was considered.

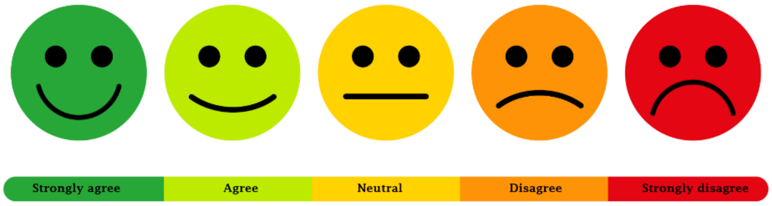

[17] Sjetne, I.S. et al. The generic short patient Experiences questionnaire (GS-PEQ): identification of core items from a survey in Norway. *BMC Health Services Research* **2011**, *11*, 88.

5. I was involved in decisions about my treatment.

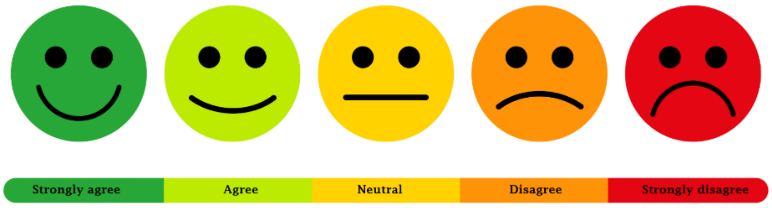

6. The consultation with the pharmacist was well-prepared and organised.

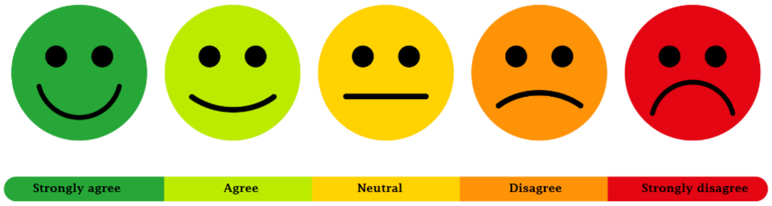

7. I am satisfied with the help I received during the medication review consultation.

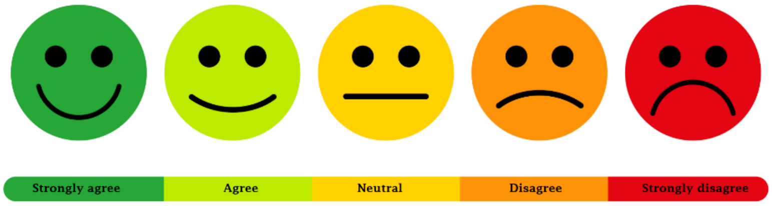

8. I benefited from the medication review consultation.

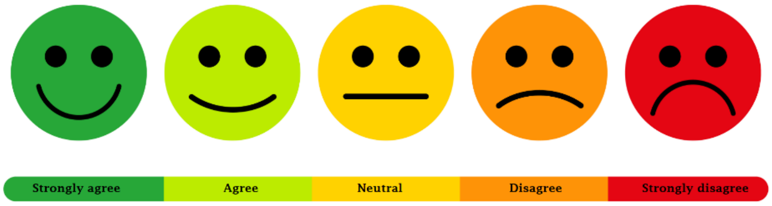

9. I recommend the medication review consultation to others.

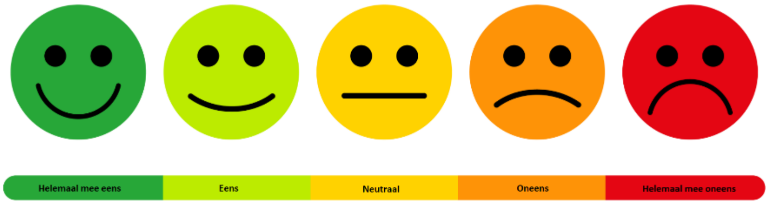

Overview S4. Three subsequent versions of the protocol for conducting MRs in Aruba, being adjusted versions of Overview S1 based on feedback received during the pilot study.

VERSION 2

1. Preparation

1.1. Patient data

Request the following patient information from the GP: episode list, active medication, stopped medication (past year), lab values, height, and weight. Remove the patient's sensitivities and contraindications from the AIS*. [*AIS = Apotheek Informatie Systeem (Pharmacy Information System)]

Copy all the data here:

1.2. Sample phone call to introduce a medication review

Preparation: during the conversation, keep the medication list next to you with the dosing frequencies and dosages.

Introduce yourself: 'I am (name), pharmacist of Botica di Servicio <name of pharmacy>.'

Ask if you're calling at the right time.

Ask if you are calling the right person (→ name, street, date of birth, AZV** number, etc.)

[**AZV = Algemene Ziektekosten Verzekering (National Ordinance General Health Insurance of Aruba)]

Explain: 'Together with my GP, I checked whether there are patients who take a lot of medicines. It is important that we check with this patient how the medication is going now. We will then check whether your illnesses are being treated properly and whether some medication needs to be added or can be removed. We would also like to see whether you, as a patient, also experience problems or wishes when it comes to medication use. This allows us to better tailor your medication use to your situation. Would you like to take a look at your medication with me?'

If the patient says no:

'That's good. If you would like to have a conversation in the future, please let us know. Then I wish you a nice day!'

If the patient says yes:

'How nice that you want to have a conversation about your medication. I will then ask a few small questions so that I can properly prepare for my conversation with you. After that, I will arrange a date and location with you. Is that okay with you?'

Other, namely:

Satisfaction about medication use	Does it have an effect? Yes/No<br>Can medication use be incorporated into your daily life? Yes/No		
Intake moments: is this going well?	When is it difficult to take the medication?		
Type of adverse events	Never	Sometimes	Often
Dizziness			
Drowsiness			
Fatigue/sleep problems			
Shortness of breath			
Muscle pain/weakness			
Bruises/bleeding			
Dry skin/itching			
Diarrhea/constipation/stomach complaints			
Dry mouth			
Problems with urinating			
Sexual problems			
Other adverse events:			

Say: 'Thank you for all the information. For the interview, it is useful if you bring all your medicines with you. This includes boxes of medicines, but also creams, puffs, painkillers, and herbal remedies. Think of paracetamol, St. John's wort, vitamins and multivitamins, and other products that you are thinking of. This gives us a complete picture of what you are using and we can advise on it.'

'Then I would like to schedule a date with you now. When would you be able to meet?'
- Date:
- Time:
- Location:

Tell the patient that there will be another email with the date, time and location and close the conversation.

Analysis

Overtreatment	
Undertreatment	
Incorrect dosage	
Dual medication	
Off-label use	
Incorrect/impractical forms of administration	
Interactions	
Contraindications	
Avoidable adverse events	
Ineffective medication	
Non-adherence	

1.3. Email to the patient after the phone call

Dear Mr/Ms/Mrs <name>,

I am <name>, pharmacist at Botica di Servicio <name of pharmacy>. We had a conversation about a medication review. In this conversation, you mentioned that you would like us to carry out this medication review. The medication review consists of a conversation with you about your medications. During this meeting, we would like to discuss your medication and wishes with you. That way, we hope to find the problems that you are experiencing. We will work together to find solutions to this.

The appointment for the medication review:

Date: XX

Time: XX

Location: XX

If the conversation takes place in the pharmacy: Don't forget to take your medication with you to the pharmacy. Think of pills, creams, puffs, and plasters. We would like to ask you to also bring products that you can buy in the drugstore without a prescription from your doctor. This helps us get a good idea of your medications.

See you soon!

Sincerely,

<name>, pharmacist at Botica di Servicio <name of pharmacy>

2. Pharmacotherapeutic anamnesis

Fill 2.1 and 2.2. (partially) during the preparation of the anamnesis.

2.1. Patient records

Name	
Age	
Address	
AZV-number	
Time of registration	
Medical specialists and other doctors who treat you	

Episode list

Episode	Start date	Medication

Lab values:

Summary of recent patient history:

Type of drug, indications, adverse events, use:

Possible adjustments:

2.2. Medication use

For the anamnesis, write down the medication with the strength and frequency and check the guidelines to see if the strength and frequency are correct. Keep track of this by highlighting the strength/frequency in red or green.

Start conversation:
Explain.
Declare professional secrecy.
Indicate that the possible action points from the conversation with the GP will be discussed.
Ask whether we as pharmacists can ask the GP about the patient's lab values.

Types of medication	Dosage	Frequency	Reason of use according to the patient + how the patient uses the medication

Herbal remedies/self-care remedies	Dosage	Frequency	Obtained from where?

NSAIDs:
Does the patient take NSAIDs? Think of ibuprofen, naproxen, diclofenac, but also Advil and Aleve for example.

Patient's goal:
What does the patient want to change/what are their goals for the conversation (if the patient doesn't understand the question, explain to the patient: think of less pain, fewer pills, more mobility)

Patient-specific questions:

Questions for the patient (write down during preparation):

2.3. Complaints

Does the medication help?
Explanation:

If you have adverse events, how much do they bother you?

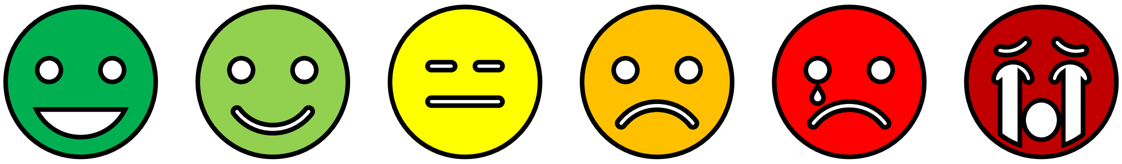

No Mild Moderate Severe Very Severe Intolerable

0 1 2 3 4 5 6 7 8 9 10

If you have multiple drug adverse events, which adverse events are the worst?

How often do you suffer from the adverse events?

Type of adverse event	Indicate how often, e.g. daily, every now and then, before bed, at night, etc.
Dizziness	
Drowsiness	
Fatigue/sleep problems	
Shortness of breath	
Muscle pain/weakness	
Bruising/bleeding	
Dry skin/itching	
Diarrhoea/constipation/stomach complaints	
Dry mouth	
Problems urinating	
Sexual problems	
Otherwise	

What grade would you give your life right now?

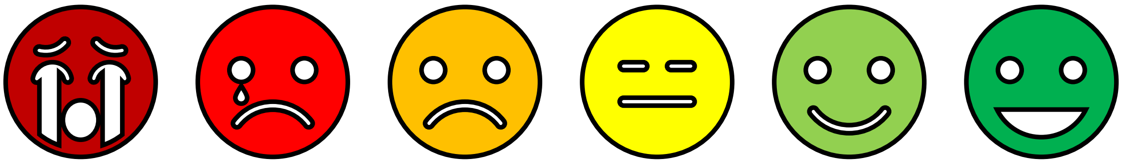

Horrible Very bad Bad Good Very good Perfect

0 1 2 3 4 5 6 7 8 9 10

Explanation:

Pain: Can you indicate how much pain you are currently in by looking at the emotions below?

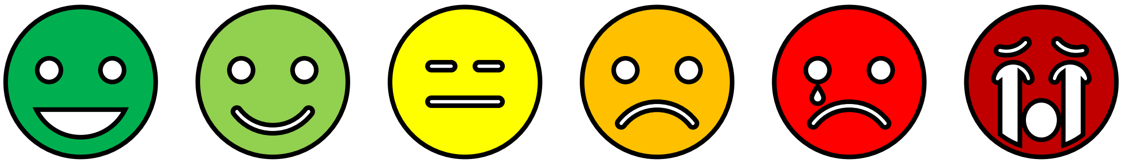

No Mild Moderate Severe Very Severe Intolerable

0 1 2 3 4 5 6 7 8 9 10

Where is the pain, when does it occur and what does that pain feel like?

Mental health: How does the patient feel? Major events? Feeling comfortable or uncomfortable, anxiety, panic, feeling depressed? Anxiety can manifest itself in: high heart rate, dry mouth, shortness of breath, tightness.[1]

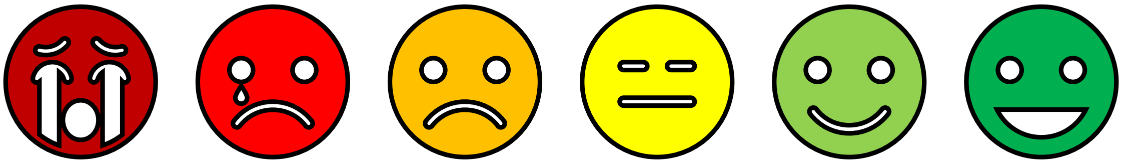

Horrible Very bad Bad Good Very good Perfect

0 1 2 3 4 5 6 7 8 9 10

Explanation:
Patient's own interpretation:

Memory/cognition: Memory problems? Think of forgetfulness, not knowing what day it is, not being able to find words, not being able to solve problems, or getting lost [2,3]
Explanation:

Morning stiffness/pain in joints
Explanation:

Label readable: Show label to patient: 'Can you read this label and explain how to use this medicine?'
Explanation:

[1]Brain Foundation. Mental health. Available from: https://www.hersenstichting.nl/mentale-gezondheid/
[2]Brain foundation. Functions of your brain. Available at: https://www.hersenstichting.nl/de-hersenen/werking-van-de-hersenen/functies/
[3]Rijnstate. Cognitive impairment. Available from: https://www.rijnstate.nl/aandoening-en-behandeling/cognitieve-stoornissen/

2.4. Practice

Daily use:
What times of the day do you take your medications?
Is it always possible to take the medication at the right time?
When do you find it difficult to take your medication?
Do you ever take someone else's medication?

Storing medicines:
Where do you store your medicines at home?
Is it also in the sight of children?

Practical problems:
Think of difficulty opening packaging, difficulty opening blisters, difficulty swallowing, difficulty using puffs, etc.

Instructional questions:
Asthma/COPD medication, creams, eye drops, insulin.

2.5. Lifestyle

Number of glasses of water/tea per day:
Daily dairy intake:
In the case of obesity: what do you eat during the day?
Number of glasses of alcohol per day (also ask specifically for beer and wine):
Smoking yes/no: If so, how often?
Drug use yes/no: If so, how often?
How long to exercise per day:
How long per day outside:

Has the patient put 3 crosses at points (in the figure below) he or she would like to work on? Then ask: 'What would you like to work on or prioritize themes in your life?'

Translation Dutch-English: movement, food, alcohol, smoking, weight, medication, family, money, work, stress, social environment, blood pressure, daily pattern, sleeping, drinking

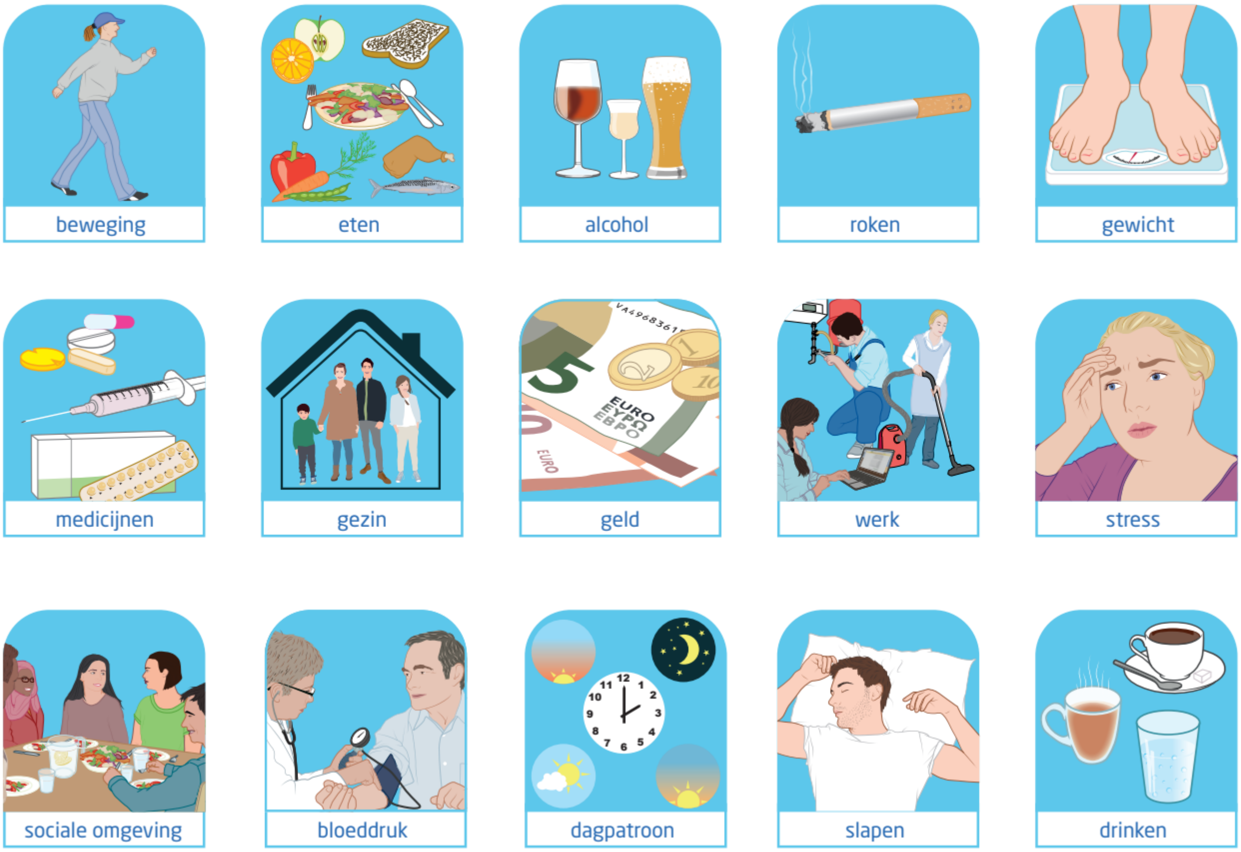

Talk to the patient: as a pharmacist, I can help with lifestyle.

What would you like help with?
Would you like a more extensive conversation about lifestyle?
Can I also share this information with your GP? Yes/no

2.6. Completion of the consultation

Provide a summary of the medication review. Mention the points that you are going to discuss with the doctor and give lifestyle recommendations where necessary. Also, ask what the patient thinks of the points you want to discuss with the doctor. If the patient does not agree with something, you do not have to discuss it with the doctor.

Additional patient notes:

3. Pharmacotherapeutic analysis

Overtreatment	
Under treatment	
Incorrect dosage	
Dual medication	
Off-label use	
Incorrect/impractical form of administration	
Interactions	
Contraindications	
Avoidable adverse events	
Ineffective medication	
Non-adherence	

3.1. Appointments, discuss later with the GP

Send the following points to the GP by email, one week before the appointment with the GP:

Proposed action points

Add	Remove	Other action point	Inquire

Action points from 'Complaints' (see pharmacotherapeutic anamnesis).
Action points from 'Practice' (see pharmacotherapeutic anamnesis).
Action points from 'Lifestyle' (see pharmacotherapeutic anamnesis).
Summary anamnesis.

Comments

Medication + dosage	Episode	Use	Action needed?	Remarks

Relevant lab values (copy from preparation).
Contraindications and sensitivities (copying from preparation).

4. Drawing up a treatment plan based on the result of the dialogue between doctor and pharmacist

4.1. Treatment plan

Date:

Action point	Execution period	Which healthcare provider does follow-up	Date of follow-up

4.2. Phone call with the patient after the appointment with the GP

Discuss all agreements that have been made with the doctor. Discuss with the patient whether he or she agrees with the agreed points. If the patient agrees, the treatment plan of 4.1 is fixed. If the patient still does not agree or if an action point is adjusted, it may be necessary to talk to the GP again.

<u>VERSION 3</u>

1. Preparation

1.1. Patient data

Request the following patient information from the GP: episode list, active medication, stopped medication (past year), lab values, height, and weight. Remove the patient's sensitivities and contraindications from the AIS*. [*AIS = Apotheek Informatie Systeem (Pharmacy Information System)]

Copy all the data here:

1.2. Sample phone call to introduce a medication review

Preparation: during the conversation, keep the medication list next to you with the dosing frequencies and dosages.

Introduce yourself: 'I am (name), pharmacist of Botica di Servicio <name of pharmacy>.'

Ask if you're calling at the right time.

Ask if you are calling the right person (→ name, street, date of birth, AZV** number, etc.)

[**AZV = Algemene Ziektekosten Verzekering (National Ordinance General Health Insurance of Aruba)]

Explain: 'Together with my GP, I checked whether there are patients who take a lot of medicines. It is important that we check with this patient how the medication is going now. We will then check whether your illnesses are being treated properly and whether some medication needs to be added or can be removed. We would also like to see whether you, as a patient, also experience problems or wishes when it comes to medication use. This allows us to better tailor your medication use to your situation. Would you like to take a look at your medication with me?'

If the patient says no:

'That's good. If you would like to have a conversation in the future, please let us know. Then I wish you a nice day!'

If the patient says yes:

'How nice that you want to have a conversation about your medication. I will then ask a few small questions so that I can properly prepare for my conversation with you. After that, I will arrange a date and location with you. Is that okay with you?'

Other, namely:

Satisfaction about medication use	Does it have an effect? Yes/No<br>Can medication use be incorporated into your daily life? Yes/No		
Intake moments: is this going well?	When is it difficult to take the medication?		
Type of adverse events	Never	Sometimes	Often
Dizziness			
Drowsiness			
Fatigue/sleep problems			
Shortness of breath			
Muscle pain/weakness			
Bruises/bleeding			
Dry skin/itching			
Diarrhea/constipation/stomach complaints			
Dry mouth			
Problems with urinating			
Sexual problems			
Other adverse events:			

Say: ‘Thank you for all the information. For the interview, it is useful if you bring all your medicines with you. This includes boxes of medicines, but also creams, puffs, painkillers, and herbal remedies. Think of paracetamol, St. John's wort, vitamins and multivitamins, and other products that you are thinking of. This gives us a complete picture of what you are using and we can advise on it.’

‘Then I would like to schedule a date with you now. When would you be able to meet?’
- Date:
- Time:
- Location:

Tell the patient that there will be another email with the date, time and location and close the conversation.

Analysis

Overtreatment	
Undertreatment	
Incorrect dosage	
Dual medication	
Off-label use	
Incorrect/impractical forms of administration	
Interactions	
Contraindications	
Avoidable adverse events	
Ineffective medication	
Non-adherence	

1.3. Email to the patient after the phone call

Dear Mr/Ms/Mrs <name>,

I am <name>, pharmacist at Botica di Servicio <name of pharmacy>. We had a conversation about a medication review. In this conversation, you mentioned that you would like us to carry out this medication review. The medication review consists of a conversation with you about your medications. During this meeting, we would like to discuss your medication and wishes with you. That way, we hope to find the problems that you are experiencing. We will work together to find solutions to this.

The appointment for the medication review:

Date: XX

Time: XX

Location: XX

If the conversation takes place in the pharmacy: Don't forget to take your medication with you to the pharmacy. Think of pills, creams, puffs, and plasters. We would like to ask you to also bring products that you can buy in the drugstore without a prescription from your doctor. This helps us get a good idea of your medications.

See you soon!

Sincerely,

<name>, pharmacist at Botica di Servicio <name of pharmacy>

2. Pharmacotherapeutic anamnesis

Fill 2.1 and 2.2. (partially) during the preparation of the anamnesis.

2.1. Patient records

Name	
Age	
Address	
AZV-number	
Time of registration	
Medical specialists and other doctors who treat you	

Episode list

Episode	Start date	Medication

Lab values:

Summary of recent patient history:

Type of drug, indications, adverse events, use:

Possible adjustments:

2.2. Medication use

For the anamnesis, write down the medication with the strength and frequency and check the guidelines to see if the strength and frequency are correct. Keep track of this by highlighting the strength/frequency in red or green.

Start conversation:
Explain.
Declare professional secrecy.
Indicate that the possible action points from the conversation with the GP will be discussed.
Ask whether we as pharmacists can ask the GP about the patient's lab values.

Types of medication	Dosage	Frequency	Reason of use according to the patient + how the patient uses the medication

Herbal remedies/self-care remedies	Dosage	Frequency	Obtained from where?

NSAIDs:
Does the patient take NSAIDs? Think of ibuprofen, naproxen, diclofenac, but also Advil and Aleve for example.

Patient's goal:
What does the patient want to change/what are their goals for the conversation (if the patient doesn't understand the question, explain to the patient: think of less pain, fewer pills, more mobility)

Patient-specific questions:

Questions for the patient (write down during preparation):

2.3. Practice

Daily use:
What times of the day do you take your medications?
Is it always possible to take the medication at the right time?
When do you find it difficult to take your medication?
Do you ever take someone else's medication?

Storing medicines:
Where do you store your medicines at home?
Is it also in the sight of children?

Practical problems:
Think of difficulty opening packaging, difficulty opening blisters, difficulty swallowing, difficulty using puffs, etc.

Instructional questions:
Asthma/COPD medication, creams, eye drops, insulin.

2.4. Complaints

Does medication help?
Yes/no, explanation:

Which adverse events do you experience most often?

Type of adverse event	Indicate how often, e.g. daily, every now and then, before bed, at night, etc.
Dizziness	
Drowsiness	
Fatigue/sleep problems	
Shortness of breath	
Muscle pain/weakness	
Bruising/bleeding	
Dry skin/itching	
Diarrhea/constipation/upset stomach	
Dry mouth	
Problems urinating	
Sexual problems	
Otherwise	

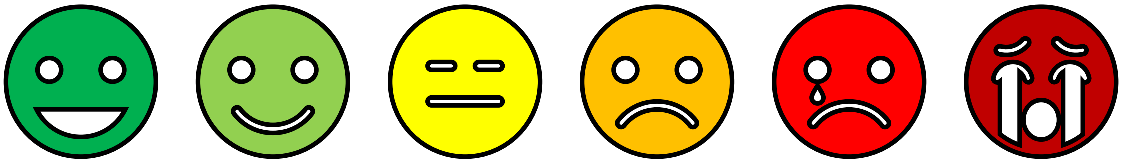

No Mild Moderate Severe Very Severe Intolerable

0 1 2 3 4 5 6 7 8 9 10

Pain: Can you indicate how much pain you are currently in by looking at the emotions below?

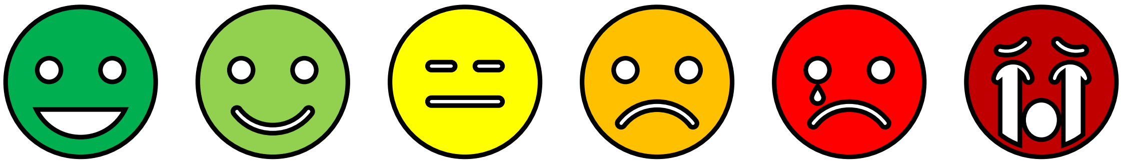

No Mild Moderate Severe Very Severe Intolerable

0 1 2 3 4 5 6 7 8 9 10

Where is the pain, when does it occur and what does that pain feel like?

How are you doing with the following topics: psychological health (think of depression, anxiety, panic), morning stiffness/pain in joints and forgetfulness

2.5. Lifestyle

Number of glasses of water/tea per day:
Daily dairy intake:
In the case of obesity: what do you eat during the day?
Number of glasses of alcohol per day (also ask specifically for beer and wine):
Smoking yes/no: If so, how often?
Drug use yes/no: If so, how often?
How long to exercise per day:
How long per day outside:

Has the patient put 3 crosses at points (in the figure below) he or she would like to work on? Then ask: 'What would you like to work on or prioritize themes in your life?'

Translation Dutch-English: movement, food, alcohol, smoking, weight, medication, family, money, work, stress, social environment, blood pressure, daily pattern, sleeping, drinking

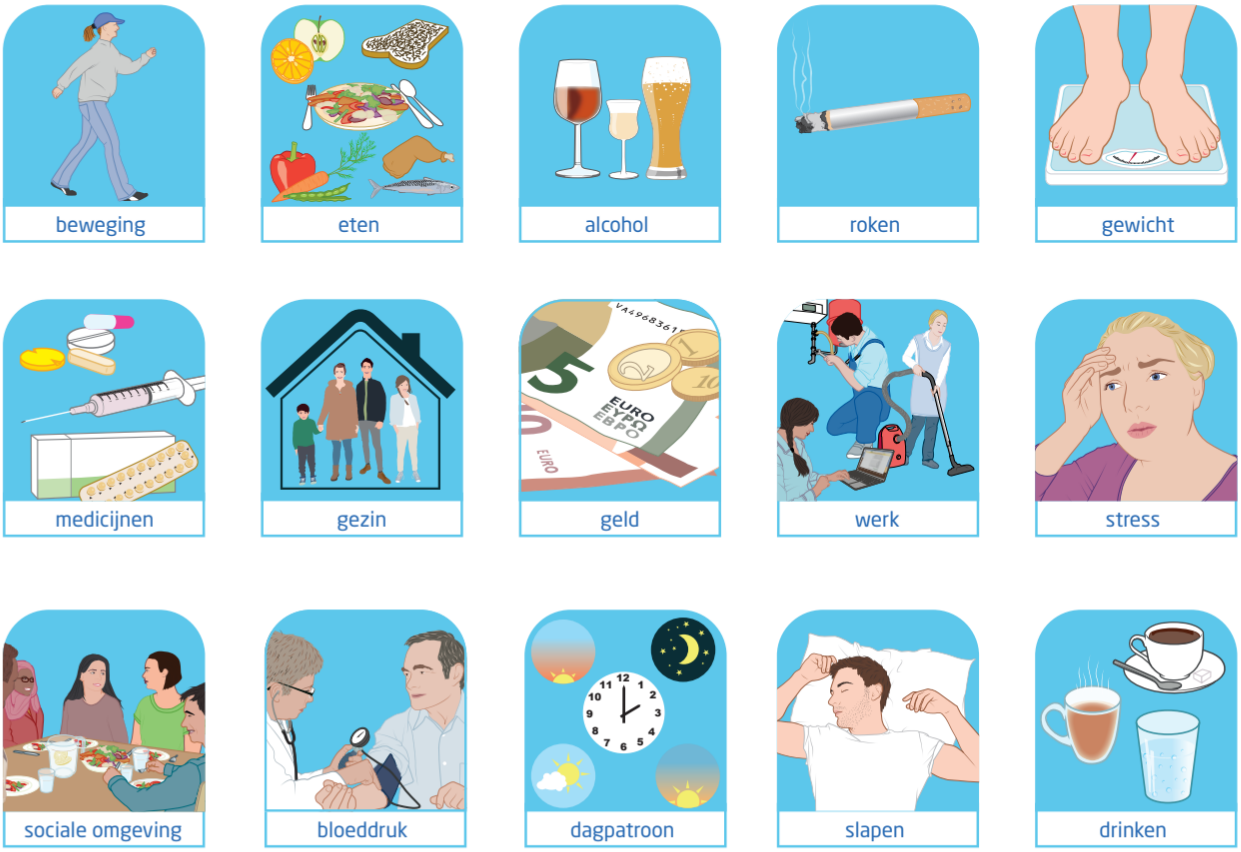

Talk to the patient: as a pharmacist, I can help with lifestyle.

What would you like help with?
Would you like a more extensive conversation about lifestyle?
Can I also share this information with your GP? Yes/no

2.6. Completion of the consultation

Provide a summary of the medication review. Mention the points that you are going to discuss with the doctor and give lifestyle recommendations where necessary. Also, ask what the patient thinks of the points you want to discuss with the doctor. If the patient does not agree with something, you do not have to discuss it with the doctor.

Additional patient notes:

3. Pharmacotherapeutic analysis

Overtreatment	
Under treatment	
Incorrect dosage	
Dual medication	
Off-label use	
Incorrect/impractical form of administration	
Interactions	
Contraindications	
Avoidable adverse events	
Ineffective medication	
Non-adherence	

3.1. Appointments, discuss later with the GP

Send the following points to the GP by email, one week before the appointment with the GP:

Proposed action points

Add	Remove	Other action point	Inquire

Action points from 'Complaints' (see pharmacotherapeutic anamnesis).
Action points from 'Practice' (see pharmacotherapeutic anamnesis).
Action points from 'Lifestyle' (see pharmacotherapeutic anamnesis).
Summary anamnesis.

Comments

Medication + dosage	Episode	Use	Action needed?	Remarks

Relevant lab values (copy from preparation).
Contraindications and sensitivities (copying from preparation).

4. Drawing up a treatment plan based on the result of the dialogue between doctor and pharmacist

4.1. Treatment plan

Date:

Action point	Execution period	Which healthcare provider does follow-up	Date of follow-up

4.2. Phone call with the patient after the appointment with the GP

Discuss all agreements that have been made with the doctor. Discuss with the patient whether he or she agrees with the agreed points. If the patient agrees, the treatment plan of 4.1 is fixed. If the patient still does not agree or if an action point is adjusted, it may be necessary to talk to the GP again.

5. Follow-up

Call the patient on the agreed dates (see 'drawing up a treatment plan') and write down what the patient has experienced.

Action point	Results and experiences according to the patient

<u>VERSION 4 (Final version)</u>

1. Preparation

1.1. Patient data

Collect the following patient data: episode list, active medication, discontinued medication (past year), laboratory values, and possibly height and weight. Ask specifically at the GP practice about the episode list, kidney function, blood pressure, LDL cholesterol, HbA1c, fasting glucose, sodium and potassium.

Copy all the data here:

1.2. Sample phone call to introduce a medication review

Preparation: during the conversation, keep the medication list next to you with the dosing frequencies and dosages.

Introduce yourself: 'I am (name), pharmacist of Botica di Servicio <name of pharmacy>.'

Ask if you're calling at the right time.

Ask if you are calling the right person (→ name, street, date of birth, AZV* number, etc.)

[*AZV = Algemene Ziektekosten Verzekering (National Ordinance General Health Insurance of Aruba)]

Explain: 'Together with my GP, I checked whether there are patients who take a lot of medicines. It is important that we check with this patient how the medication is going now. We will then check whether your illnesses are being treated properly and whether some medication needs to be added or can be removed. We would also like to see whether you, as a patient, also experience problems or wishes when it comes to medication use. This allows us to better tailor your medication use to your situation. Would you like to take a look at your medication with me?'

If the patient says no:

'That's good. If you would like to have a conversation in the future, please let us know. Then I wish you a nice day!'

If the patient says yes:

'How nice that you want to have a conversation about your medication. I will then ask a few small questions so that I can properly prepare for my conversation with you. After that, I will arrange a date and location with you. Is that okay with you?'

If the patients says yes, you can ask questions about the following points (add notes where necessary and thick what has been asked).

Satisfaction about medication use	Does it have an effect? Yes No<br>Can medication use be incorporated into daily life? Yes No		
Intake moments: is this going well?	When is it difficult to take the medication?		
Suffering from adverse events?	Yes/no. Explanation:		
Frequency of adverse events?	Never	Sometimes	Often

Say: ‘Thank you for all the information. For the interview, it is useful if you bring all your medicines with you. This includes boxes of medicines, but also creams, puffs, painkillers, and herbal remedies. Think of paracetamol, St. John's wort, vitamins and multivitamins, and other products that you are thinking of. This gives us a complete picture of what you are using and we can advise on it.’

‘Then I would like to schedule a date with you now. When would you be able to meet?’
- Date:
- Time:
- Location:

Now close the call.

2. Pharmacotherapeutic anamnesis

Fill 2.1 and 2.2. (partially) during the preparation of the anamnesis.

2.1. Patient data

Name	
Age	
Address	
AZV-number	
Time of registration	
Medical specialists and other doctors who treat you	

Episode list of the GP

Episode	Start date	Medication

Patient’s medication history:

Lab values:

Contraindications according to pharmacy:

Summary of patient's recent history (if GP has provided the history of consultations):

Type of drug, indications, adverse events, use:

Possible adjustments:

2.2. Medication use

For the anamnesis, write down the medication with the strength and frequency and check the guidelines to see if the strength and frequency are correct. Keep track of this by highlighting the strength/frequency in red or green.

Start conversation:
Explain.
Declare professional secrecy.
Indicate that you, as a pharmacist, want to have an open and honest conversation.
Indicate that possible action points from the conversation with the GP will be discussed.
Ask if the pharmacist is allowed to ask the patient's GP for lab values.

Medication	Dosage	Frequency	Reason for patient use + method of use (dosage, frequency)

Herbal remedies/self-care remedies	Dosage	Frequency	Obtained from where?

Hospital:
Does the patient receive medication from the hospital?

NSAIDs:
Does the patient take NSAIDs? Think of ibuprofen, naproxen, diclofenac, but also Advil and Aleve for example.

Patient's goal:
What does the patient want to change/what are their goals for the conversation (if the patient doesn't understand the question, explain to the patient: think of less pain, fewer pills, more mobility)

What kind of questions does the patient have?

Questions for the patient (write down during preparation):

2.3. Practice

Daily use:
At what times of the day do you take your medications?
When do you find it difficult to take your medication?
Do you ever take someone else's medication?
Label readable: Explanation, show label to patient: 'Can you read this label and explain how to use this medicine?'

Storing medicines:
Where do you store your medicines at home?
Is it also in the sight of children?

Practical problems:
Think of difficulty opening packaging, difficulty opening blisters, difficulty swallowing, difficulty using puffs, etc.

Instructional questions:
Asthma/COPD medication, creams, eye drops, insulin.

2.4. Complaints

Does medication help?
Explanation:

Which adverse events bother you most?

Type of adverse event	Indicate how often, e.g. daily, every now and then, before bed, at night, etc.
Dizziness	
Drowsiness	
Fatigue/sleep problems	
Shortness of breath	
Muscle pain/weakness	
Bruising/bleeding	
Dry skin/itching	
Diarrhea/constipation/upset stomach	
Dry mouth	
Problems urinating	
Sexual problems	
Otherwise	

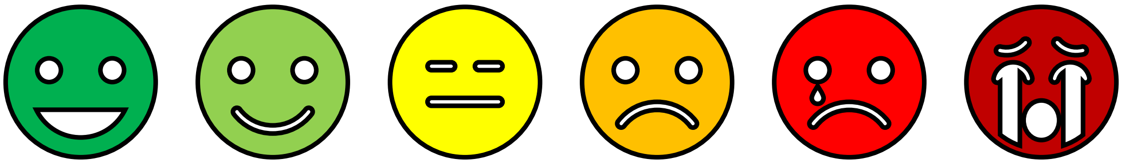

No Mild Moderate Severe Very Severe Intolerable

0 1 2 3 4 5 6 7 8 9 10

Pain: Can you indicate how much pain you are currently in by looking at the emotions below?

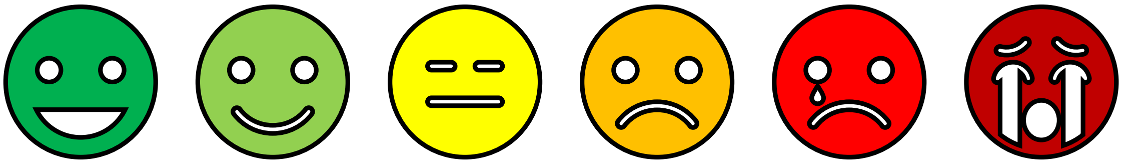

No Mild Moderate Severe Very Severe Intolerable

0 1 2 3 4 5 6 7 8 9 10

Where is the pain, when does it occur and what does that pain feel like?

How are you doing with the following topics: psychological health (think of depression, anxiety, panic), morning stiffness/pain in joints and forgetfulness

2.5. Lifestyle

Number of glasses of water/tea per day:
Daily dairy intake:
In the case of obesity: what do you eat during the day?
Number of glasses of alcohol per day (also ask specifically for beer and wine):
Smoking yes/no: If so, how often?
Drug use yes/no: If so, how often?
How long to exercise per day:
How long per day outside:

Has the patient put 3 crosses at points (in the figure below) he or she would like to work on? Then ask: 'What would you like to work on or prioritize themes in your life?'

Translation Dutch-English: movement, food, alcohol, smoking, weight, medication, family, money, work, stress, social environment, blood pressure, daily pattern, sleeping, drinking

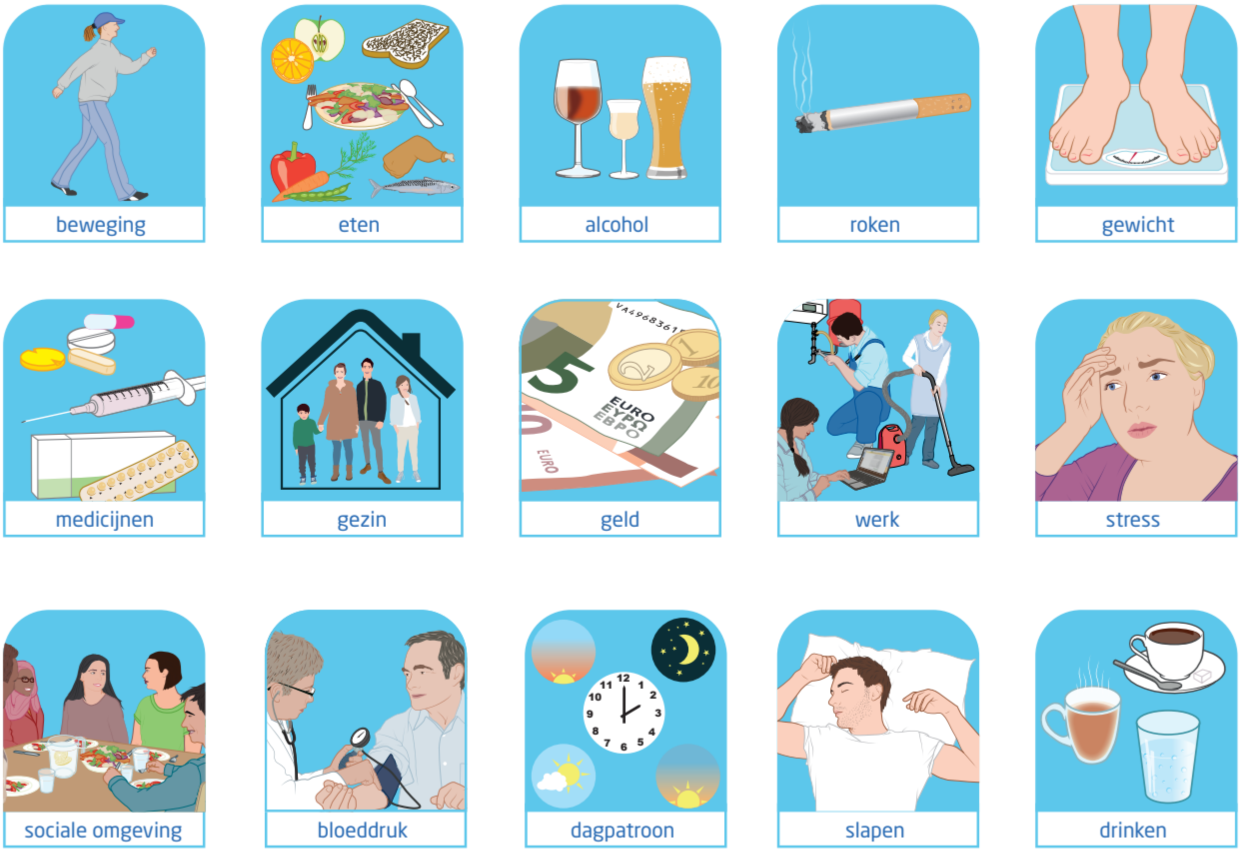

Talk to the patient: as a pharmacist, I can help with lifestyle.

What would you like help with?
Would you like a more extensive conversation about lifestyle?
Can I also share this information with your GP? Yes/no

2.6. Completion of the consultation

Provide a summary of the medication review. Mention the points that you are going to discuss with the doctor and give lifestyle recommendations where necessary. Also, ask what the patient thinks of the points you want to discuss with the doctor. If the patient does not agree with something, you do not have to discuss it with the doctor.

Additional patient notes:

3. Pharmacotherapeutic analysis

Overtreatment	
Under treatment	
Incorrect dosage	
Dual medication	
Off-label use	
Incorrect/impractical form of administration	
Interactions	
Contraindications	
Avoidable adverse events	
Ineffective medication	
Non-adherence	

3.1. Appointments, discuss later with the GP

Send the following points to the GP by email, one week before the appointment with the GP (please note, also check the purchase prices for new medication proposals!):

Proposed action points

Add	Remove	Other action point	Inquire

Action points from 'Complaints' (see pharmacotherapeutic anamnesis).
Action points from 'Practice' (see pharmacotherapeutic anamnesis).
Action points from 'Lifestyle' (see pharmacotherapeutic anamnesis).
Summary anamnesis.

Overview of active medication and corresponding episode

Medication + dosage	Episode	Use	Action needed?	Remarks

Relevant lab values (copy from preparation).
Contraindications and sensitivities (copying from preparation).

4. Drawing up a treatment plan based on the result of the dialogue between doctor and pharmacist

4.1. Treatment plan

Date:

Action point	Execution period	Which healthcare provider does follow-up	Date of follow-up

4.2. Phone call with the patient after the appointment with the GP

Discuss all agreements that have been made with the doctor. Discuss with the patient whether he or she agrees with the agreed points. If the patient agrees, the treatment plan of 4.1 is fixed. If the patient still does not agree or if an action point is adjusted, it may be necessary to talk to the GP again.

5. Follow-up

Call the patient on the agreed dates (see 'drawing up a treatment plan') and write down what the patient has experienced.

Action point	Results and experiences according to the patient

Processing results: add this overview to the patient file and send an overview to the GP by email.

6. Processing patient file

Medication review (name, date of birth), elaboration of MR, and proposed action points to GP.

Proposed action points:

Add	Remove	Other action point	Inquire

Action points from 'Complaints' (see pharmacotherapeutic anamnesis).

Action points from 'Practice' (see pharmacotherapeutic anamnesis).

Action points from 'Lifestyle' (see pharmacotherapeutic anamnesis).

Summary anamnesis.

Overview of active medication and corresponding episode:

Medication and dosage	Episode	Use	Action needed?	Remarks

Relevant lab values (copy from preparation).

Contraindications and sensitivities (copying from preparation).

Date follow-up:

Action point	Execution period	Which healthcare provider does follow-up	Date of follow-up

Passing on to the patient: (write here what the GP and what exactly the pharmacist will pass on to the patient after drawing up the treatment plan).

(date) – call patient: (write down here what you have explained to the patient).

Send this overview to the GP and scan it for the patient file. If necessary, this overview can be supplemented at a later date with the results of the follow-up.
